# Factors influencing malnutrition among adolescent girls in The Gambia: a mixed-methods exploratory study

**DOI:** 10.1186/s12889-024-21242-w

**Published:** 2025-01-08

**Authors:** Haddy Jallow-Badjan, Tanefa A. Apekey, Maria J. Maynard

**Affiliations:** 1 Migrant Health Research Group, School of Health, Leeds Beckett University, Leeds, UK; 2 Sheffield Centre for Health and Related Research, University of Sheffield, Sheffield, UK

**Keywords:** Female Adolescents, Malnutrition, Social Determinants of Health, Gambia

## Abstract

**Background:**

In The Gambia, existing research to understand and address malnutrition among adolescent girls is limited. Prior to the conduct of large-scale studies, formative research is needed. The aim of this mixed methods, cross-sectional study was to explore cultural contexts relevant to nutritional status, feasibility and appropriateness of recruitment and data collection methods (questionnaires and anthropometric measures), and plausibility of data collected.

**Methods:**

The study took place in May–June 2021 in an urban conurbation in Brikama local government area (LGA) and two rural villages in Mansakonko LGA, The Gambia. The purposive sampling frame of the all-female sample included residence in the selected urban or rural settings and being aged 10–14 or 15–19 years. Thirty-two girls aged 10 to 19 years, with equal numbers in urban and rural settings were recruited. Four focus groups discussions (FGDs), with eight participants in each, were held to understand perspectives on cultural practices; concepts of under- and overweight, and research recruitment methods. The same participants completed questionnaires on socioeconomic circumstances, health, access to community resources, nutrition knowledge, sleep, and physical activity, and had anthropometric measures taken. FGDs were then reconvened to discuss the feasibility and acceptability of the questionnaires and anthropometric measures, and views on providing biological samples in the future. FGD data were analysed using thematic analysis. Body mass index (BMI)-for-age and height-for-age z-scores, mid-upper arm circumference, and waist: hip ratios were assessed and descriptive statistics used to explore the data obtained.

**Results:**

Five themes were identified in the focus group discussions: 1. Cultural norms: harmful vs. beneficial to nutrition-related health; 2. Concepts of healthy diet and weight; 3. Approaches to tackling under- and overnutrition; 4. Study recruitment: barriers and facilitators; 5. Study questionnaires and proposed measures are mostly feasible and acceptable. Questionnaire data highlighted limited access to resources (e.g. food markets and electricity) as important individual, household and community factors influencing malnutrition in rural settings. The anthropometric measures reflected the double burden of malnutrition in The Gambia, with the presence of stunting (41%), underweight (31%), and living with overweight or obesity (10%). A higher proportion of participants were underweight in rural compared to urban settings (50% vs 12.5% respectively, *p* = 0.03). Over 70% of those classified as underweight perceived their weight as normal.

**Conclusion:**

This exploratory study provides novel data to inform larger-scale research to understand and address malnutrition among adolescent females in The Gambia. Urban–rural variance in the double burden of malnutrition, factors influencing malnutrition, and in the barriers to and facilitators of adolescents taking part in research, are key considerations.

**Supplementary Information:**

The online version contains supplementary material available at 10.1186/s12889-024-21242-w.

## Background

In The Gambia, malnutrition in all its forms (i.e. undernutrition [underweight, stunting, wasting, and/or micronutrient deficiency] and overnutrition [overweight and obesity]) is a significant public health problem [1]. As with other age groups in The Gambia, undernutrition co-exists with rising overnutrition among adolescents (i.e. young people aged 10–19 years) [2–4]. It has been reported that girls in The Gambia are also more vulnerable to underweight, overweight and obesity than boys [2, 4], and therefore specific attention on female adolescents is warranted. Improving nutrition during this period in the life course has the potential benefits of optimising contemporary physical, psychological and cognitive health, long-term health, and the development and health of future generations [5]. Socioecological models (SEM) for understanding and addressing health behaviours and behaviour change emphasise the interactions across individual, household, community and national levels [6]. There is limited existing research which considers these multiple levels of influence on nutrition-related health among adolescent girls in The Gambia. Available evidence in low and middle income countries (LMICs) more widely, implicates a range of individual- and household-level factors in the prevalences of underweight or stunting [7, 8], overweight and obesity [9, 10], and nutrient deficiencies and associated health outcomes such as in iron deficiency anaemia [8]. The available literature has a number of limitations, including providing sparse evidence overall (with none in The Gambia examining multiple levels of influence on nutrition), and limited focus on the wider determinants of malnutrition [11].

The Gambia has a young population with 220,980 (23%) aged 10 to 19 years [12]. National data shows that the trend of undernutrition among girls and women aged 15–49 years has improved from 17% in 2013 to 14% in 2019–20 but is still highly prevalent, whilst overweight and obesity has worsened with a prevalence of 23% in 2013 rising to 36% in 2019–20 [1]. Girls and women in this age group living in rural areas of The Gambia are more likely to be underweight than urban females (17% vs 13%) whilst the opposite is true for overweight and obesity with higher prevalence in urban areas (40% vs 25%). The prevalence of anaemia among 15–49 year-olds has decreased in the country from 60% in 2013, but remains high at 44%, according to the most recent national data [1]. Where adolescent age-specific national data are available (for cross-sectional data, but not for trends) this indicates that 28% of 15–19 year-olds are underweight, 12% are overweight or obese, and 43% have anaemia [1]. Previous research focusing on adolescents in The Gambia (mean age 16.8 years) has shown that almost 14% of secondary school-going youths (males and females combined) were underweight at the same time as more than 7% being either overweight or obese, thus indicating the double burden of malnutrition [2]. Factors associated with nutritional status were female sex, nighttime sleep duration, mother’s education, and physical activity levels. Malnutrition-related research including adolescent girls therefore does exist in The Gambia, as highlighted above. However, either the full age span of adolescence (10–19 years) was not covered, or gender- and age- specific analyses were not reported. Also, previous studies conducted in the country have focused on individual-level determinants of adolescent girls’ nutrition-related health, and the role of wider determinants remains to be elucidated. Malnutrition is a complex issue and multicomponent coproduced interventions are needed to address malnutrition among adolescents in The Gambia [13]. The period of preparation prior to and during intervention development may require several small studies, including exploratory research with future intervention beneficiaries and other stakeholders [14]. Based on the available literature, and to the authors’ knowledge, no previous study conducted in The Gambia has considered the individual, household, community, and national determinants of adolescent girls’ nutritional status. As the first of its kind in the country, an exploratory study was imperative to understand the cultural context in which the research is situated and the feasibility of conducting further observational or interventional research within the target population. Thus, the overall aim of this study was to conduct formative research exploring cultural norms, views on engaging in research, and the survey methods used to assess the nutritional status of adolescent girls aged 10–19 years, living in urban and rural areas of The Gambia. Specific objectives were to: 1. Use focus group discussions to: (i) Gain understanding of the cultural contexts relevant to nutritional status (cultural norms and practices; concepts of healthy diet and weight; prevention and treatment of under- and overweight); (ii) Explore views on research approaches that may enhance a larger study (optimal recruitment methods; feasibility and acceptability of self-reported questionnaires and anthropometric measures); 2. Assess the quality and plausibility of the data (i) obtained from the questionnaires (health, diet, physical activity, potential individual-level and wider [household; community] influences on nutritional status), and (ii) nutritional status outcomes derived from the anthropometric measures (weight status, abdominal obesity, and stunting); 3. Where possible, explore similarities and differences between participants in urban and rural settings within objectives 1 and 2; and 4. Identify any required adaptations to optimise the data collection tools and procedures for larger-scale research.

## Materials and methods

### Conceptual framework

**Fig. 1:**
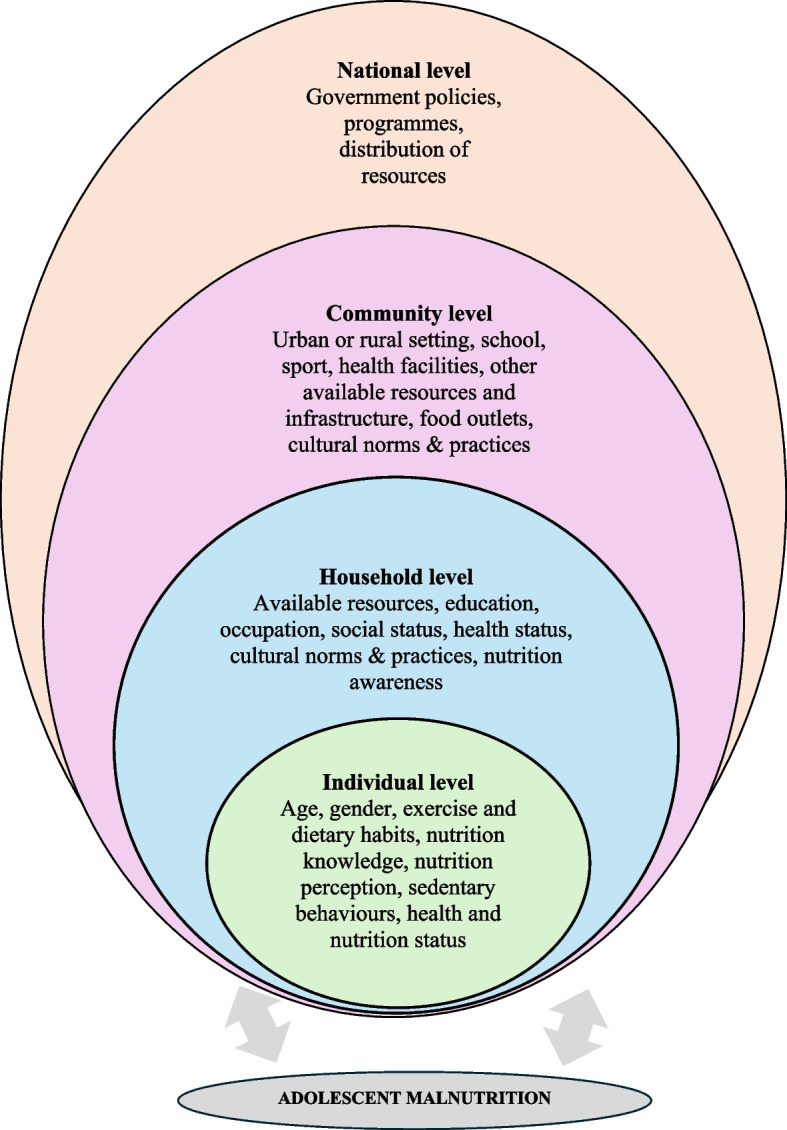
Conceptual framework

The study was conducted as part of a wider programme of research [15], underpinned by a conceptual framework informed by SEM [6]. Variations on the levels of influence and their labels depicted in models nevertheless retain an emphasis on interactions between levels of influence, and on the notion that behaviours are influenced by the social environment [6]. The levels of influence (individual, household, community, national) and factors of interest within levels are depicted in Fig. 1. In the phase of the research reported here, individual level (by self-report questionnaire and physical measures), household and community levels (self-report questionnaire and focus group discussion) and policy levels (focus group discussion) of influence could be explored, largely from adolescents’ own perspectives. However, some elements of the framework (e.g. measured dietary habits at the individual level), policy analysis and views of policy actors with regard to adolescent malnutrition were conducted in subsequent phases of the research to be reported at a later date.

### Study design

A mixed methods, cross-sectional study design was used in this exploratory study. Qualitative approaches aid understanding of the meanings people attach to actions, decisions, beliefs and values within their social worlds [16]. Exploring the quantitative study elements provides preliminary data on the feasibility of research protocols and the efficacy of instruments, reducing research waste [17]. Gaining knowledge using qualitative and quantitative approaches informs the design, acceptability and delivery of large scale observational studies [17] and person-centred health programmes [18]. Transparent reporting was aided by two checklists suitable for the study design: the consolidated criteria for reporting qualitative studies (COREQ) [19] and the STrengthening the Reporting of OBservational studies in Epidemiology (STROBE) checklist for cross-sectional studies [20] (Supplementary File 1). Qualitative focus group discussions (FGDs) were conducted to examine cultural norms and practices that could influence adolescent girls’ nutritional status, and elements of study design that are important to the feasibility of future studies (objective 1). Quantitative data were collected via questionnaire and anthropometric measures (objective 2), with urban and rural comparisons across the datasets made where possible (objective 3). Both the quantitative and qualitative data were used to identify any required adaptations needed to optimise the data collection tools and procedures (objective 4), informing the subsequent larger survey and intervention development research. The study was conducted by the lead author (HJB) [15], supervised by the co-authors (MJM; TAA). Data collection was supported by field workers trained and managed by HJB (the ‘research team’; see Data collection personnel, below).

### Study setting

The research settings were urban and rural areas in The Gambia. Brikama local government area (LGA) is the most populated region in the country and is comprised of 80% urban settlements; Mansakonko LGA was selected as the rural site [21]. As the urban settlements are more densely populated than rural areas, one urban settlement and two rural villages were selected for the study to achieve equal population representation. The largest ethnic group (i.e. tribe) in Brikama are Mandinka (39.8% of the Brikama population), followed by Fula (19.7), Jola (18.6%), Wolof (10.7%) and other tribes (11.1%) [22]. A typical urban setting (a busy commercial hub with a wide range of amenities) was identified in Brikama LGA, hereafter referred to as the ‘urban area’. Almost 80% of the population in the Mansakonko region identify as being Mandinka, followed by Fula (16.2%), Jola (2.4%), and other tribes (1.3%) [23]. There are 36 villages in the region and the primary occupation is subsistence farming. Two rural areas (referred to as ‘village 1’ and ‘village 2’ or the ‘rural villages’, as appropriate) were identified as classic village environments in Mansakonko LGA. The research settings are predominantly Muslim societies, which resonates with the national picture [22].

All procedures were conducted in accordance with the Declaration of Helsinki. Ethical approval for the study was granted by the Research Ethics Committee, School of Health, Leeds Beckett University (Reference: 73,304), and The Gambia Government/MRCG Joint Ethics Committee (Reference: R020021). Permission was also sought from Chiefs (known as *Alkalos*), village and compound heads in their respective regions. Consent from parents of participants who were under 18 years and from adolescents over 18 years, and assent of the participants was certified by the completion and signing of consent and assent forms.

### Sampling and recruitment

A purposive (or purposeful), non-probability sampling technique was used. Stratified purposive sampling involved identifying different strata or categories within the population of interest, whereby participants were relatively homogenous within categories [24]. Purposefully recruiting a range of participants within strata incorporates what is known as maximum variation sampling within the stratified approach [24]. Maximum variation aids documenting a variety of views, and identifies important patterns in the phenomena of interest that are common across the diversity of the sample [24]. The sampling categories and focus group composition are shown in Table 1. Categories included younger (aged 10–14 years) and older (aged 15–19 years) adolescent girls, resident within the urban Brikama LGA or the rural Mansakonko LGA. A minimum of 4 and maximum of 8 participants in each focus group was the aim; therefore, the total target sample size was 16–32 adolescent girls. The small but diverse sample provided ‘information-rich cases for in-depth study’ ( [24];p.264). Further, it has been postulated that 4–8 focus groups are sufficient to achieve saturation of the themes identified in the data [25].

**Table 1:** Stratified focus group purposive sampling frame

Setting/ focus group	Aged 10–14 years	Aged 15–19 years	Total
Urban area (urban focus group 1)	2–4	2–4	4–8
Urban area (urban focus group 2)	2–4	2–4	4–8
Rural village 1 (rural focus group 1)	2–4	2–4	4–8
Rural village 2 (rural focus group 2)	2–4	2–4	4–8
**Total target sample size**:			**16–32**

The focus group participants also completed the questionnaires and physical measures. By design, although the questionnaire and measures captured quantitative data, the aim was not statistically representative data. The emphasis was on exploring feasibility and acceptability of the measures and the quality and plausibility of the data, as detailed in the objectives. Due to the non-probability nature of the sampling, it is not possible to know how many potentially eligible participants were made aware of the study, and thus a response rate cannot be calculated.

Building relationships with focal persons in each of the communities was an important element of reaching potential participants, and the recruitment process is further outlined below.

#### Engagement and recruitment in the rural areas

It was relatively straightforward to engage with the rural communities (compared to the urban setting, see below) due to the common existence of organised groups such as the Village Development Committee (VDC), tasked with the welfare of its members. Thus, it was possible to identify the VDC chairman in each of the villages, well known and highly regarded in their communities, who acted as focal persons. Recruitment visits were made to both villages from 24th to 27th May 2021. In village 1, the meeting was held at the *Alkalo’s* compound with 35 village elders (both men and women) present. The session was very interactive and it was unanimously stated that consent would not be given for their children to participate if COVID vaccination, which they did not trust, was part of the study. There was also concern about girls becoming anaemic because of the blood samples planned for future phases of the study. Explanations that the project was not investigating anything related to COVID, that the research would not include any vaccinations, and that the amount of blood that would be collected in future phases of the research (typically 8mls per person) would not cause harm to the participants were accepted. The community leaders indicated that they felt reassured, and that recruitment and taking consent could be done that day. Any parent present at the meeting with their child (or whose child was playing nearby) and interested in their child participating consented before leaving. At village 2, the research team met the *Alkalo* at his compound and some of the elders, facilitated by the VDC chairman. The elders welcomed the focus on adolescents but raised concerns about their community being accessed by previous research groups with impact yet to be seen in the community. However, approval was granted at the end of the discussion. The *Alkalo* and elders spoke with parents/ guardians of eligible adolescents. Where interest was registered, researchers were invited to the respective homes, met with the prospective participants and their parents/guardians and the consent procedures were carried out. Dates in June 2021 were agreed for the researchers to return to both villages for data collection.

#### Engagement and recruitment in the urban area

Engagement was a little more challenging in the urban area. The large geographical area meant that more than one focal person was required in different parts of the community, and there was less openness to non-residents, compared to the rural areas. Recruitment took place during 28th May to 10th June 2021 and included multiple consultations, telephone calls and meetings with key informants, identification of four focal persons resident in the selected urban area, and several visits to meet potential participants’ parents/ guardians. Parents/ guardians then discussed the project with their children, and for those registering interest, the researchers were invited to their individual homes where potential participants were met. Parental approval was granted, consent/ assent and recruitment confirmed, and a date agreed for later in June 2021 for the data to be collected at the compound of one of the focal persons.

### Focus group discussions

A semi-structured interview guide was developed (Supplementary File 2). The topics were informed by the study objectives, research conducted in The Gambia by the lead author [2, 4] and consequent experience of working with the target group, and previous studies conducted in other LMICs [7, 8, 11, 26, 27]. The topics included optimal enrolment methods, perceptions of healthy diet and weight, cultural norms and practices, opinions on appropriate solutions to adolescent girls’ malnutrition, opinions on the acceptability of blood and urine sample collection, potential techniques for longitudinal follow up of participants, and views on the questionnaire.

### Questionnaire and anthropometric measures

#### Questionnaire

The questionnaire comprised of six sections (Supplementary File 3). All sections were informed by the objectives and the relevant sections of questionnaires used among adolescents in The Gambia by HJB [2, 4]. Previously used sections were of bespoke design for the setting, except with regard to the physical activity questions which were based on an adapted existing tool (see below). The first part of the questionnaire included individual and household demographic and socioeconomic questions (age, country of birth, religion, school attendance, grade, marital status, number of siblings, family type and home ownership, parental occupation and education level, and source of drinking water), followed by questions on participants’ maturation status and health (menstruation, parity, malaria, treatment for parasitic worms). Further sections assessed access to community resources (food markets, health services, roads, electricity, and schools), sources of nutrition knowledge (family, peers, school, mass media, community members and healthcare practitioners), physical activity, sleep and sedentary activity. The Physical Activity Questionnaire for Adolescents (PAQ-A) [28] was incorporated into the questionnaire. As in previous use in The Gambia [2] and in similar settings among South African adolescents [29], activities that were not relevant were removed (such as hockey, skiing and ice-skating).

##### Questionnaire validity

Critique of the questionnaire in the FGDs was a key element of the qualitative assessment of questionnaire face and content validity [30]. Additionally, the recommendations made by the adolescents were evaluated by the research team with regard to difficulty or ambiguity, choice of wording and ordering of items, and decisions made about any changes required to the questionnaire. The PAQ-A tool used to measure physical activity has been shown to have high reliability based on the intra-class correlation coefficient (*r* = 0.719, *p* < 0.05; 95% confidence interval (CI): 0.569–0.822) and fair convergent validity (*r* = 0.516, *p* < 0.05) when previously validated among youth [31]. Any within-study assessment of quantitative construct validity was precluded by small sample size and/ or predominantly nominal, categorical data.

#### Anthropometric measures

The measurements taken included weight, height (from which BMI and BMI for age z-scores were calculated, see below), mid upper arm circumference (MUAC), and waist and hip circumferences (from which waist-hip-ratio (WHR) was derived, see below). In addition to standing height, demi-span and ulna length measures were also conducted as recommended alternatives when standing height is not feasible due to illness or disability [32–35]. Standardised protocols based on WHO guidance [36] were followed for all measurements. A digital scale (Seca 869) was used to measure weight and a stadiometer (Seca 217) to measure standing height. UNICEF MUAC measuring tapes were used for mid upper arm circumference (MUAC), and simple measuring tapes were used for waist and hip circumferences, demi-span and ulna lengths.

### Data collection personnel

Data collection was led by the lead author (HJB), a Gambian woman, nurse, and a doctoral student at the time of the study (now a post-doctoral researcher). HJB has over 10 years experience as a researcher and health professional working with the target populations, and is multi-lingual (English, Mandinka, Fula, and Wolof). Led and trained by HJB, eight research assistants (RAs), four women and four men, assisted in the data collection process. These were nursing students in their final year of training or graduates who had recently completed their degree programme. All were also fluent in English, Mandinka (the main spoken language in Mansakonko), Fula and Wolof. The collective language skills meant that interactions with the participants during data collection could be in the preferred language/s of the participants. In addition to the RAs knowledge of malnutrition gained as part of their nurse training programme, HJB provided a one-day training session in May 2021 for the measurement techniques, reading through the questionnaire, and overall format of the data collection process. Prior to the training day, a meeting with the RAs was held where the aim of the research programme was explained, and the data collection stages and strategies were discussed. They were also provided with the data collection tools such as the interview guide, questionnaire, anthropometric measurement protocols, participant information sheet, consent and assent forms and recruitment posters. These were given in advance so that the RAs could read through the content and clarify queries on the day of the training exercise. The three sections of the training day included 1. Review and discussion of the focus group interview guide, and the roles of note taking, checking the recording device, and ways of ensuring participants’ comfort to support HJB in facilitating the FGDs; 2. Discussion of the questionnaire during which the RAs were encouraged to raise queries, critique questions, and make suggestions for revisions; and 3. Reviewing the anthropometry protocols, together with demonstrations of all the measurements by the lead researcher. Each RA then had the opportunity to practice each measurement at least three times, working in pairs. The RAs retained the data collection tools for further familiarisation in their own time.

### Data collection procedures

The data collection process commenced with the FGDs. Two FGDs were conducted in the urban area and two in the rural villages (one FGD in each village), with a total of eight participants in each group. RAs were allocated to a specific FGD for note taking, together with the lead researcher who acted as facilitator, which prevented overwhelming the participants with too many people at the same time. Participants were only known to the research team in as far as their presence at the activities raising awareness of the project (described above). Confidentiality was discussed and the written permission to conduct and record the sessions was further verbally confirmed before commencing. All participants were reminded that they could stop taking part in the sessions at any time. The FGDs were shaped by the interview guide, with probing where needed. Questionnaire completion followed the FGD with participants sat at least two metres apart to maintain confidentiality and to prevent them discussing their answers. The anthropometric measurements were then taken. In each setting, a station was created where all measurements were conducted, with screened areas for privacy. The station was divided into two sub stations with one researcher conducting the measurement while the other recorded the results. Anthropometric measurements were taken three times and the average was calculated as the final value. Only one participant at a time was permitted to attend a sub-station to ensure privacy and they had the option of having a female conduct their measurements if preferred. After completion of the measurements, the FGD was then resumed for the final session to discuss the questionnaire. Breaks were taken between sessions.

In village 1, the FGD was conducted in one of the *Alkalo’s* neighbouring compounds, lasting 1 h and 43 min. Overall, the data collection process lasted just under five hours, including breaks. In village 2, the VDC chairman requested that the FGD take place at community centre meeting point (known as a *Bantaba*). The FGD lasted 1 h 19 min, and the data collection session took about four hours overall. The FGDs in the urban setting lasted for 1 h 45 min and 1 h 31 min, respectively. In total, the data collection for each of the two urban sessions lasted around 3 h 30 min.

### Qualitative data management and analysis

The qualitative data were analysed using The Framework Method to generate topic summaries guided by the research objectives [37]. The analysis was positioned within a realist stance that centred participants subjective views but also encompassed underlying realities [38]. Analytical stages included verbatim transcription, followed by data familiarisation through reading and re-reading transcripts and re-listening to the audio recordings. Initial code generation was followed by developing the analytical framework. Applying the analytical framework, identifying initial themes, charting, and interpreting data were the last stages of the process. A defining feature of The Framework Method is the matrix output which consists of rows (cases), columns (codes) and ‘cells’ of summarised data, providing a structure into which the data can be analysed (the framework) by case and by code [37]. In the current study, after hand coding the transcripts in MS Word, cases, codes, and relevant data from the transcripts were transferred into MS Excel for charting and identifying themes. Saturation was determined by iterative analysis alongside data collection, and the charting of the anonymised participants and themes generated in MS Excel, in line with the Framework Method [37]. Five overarching themes were identified (see results) and saturation of themes was deemed to have occurred after four focus groups, although cannot be guaranteed [39]. Presentation of the themes in the results section is supported by direct quotes from the data labelled with participant’s ID, rural or urban setting, and participant’s age.

Rigour in the analysis was ensured by clarity in the methods and data analysis. Systematic analysis included constant comparison between cases, paying attention to ‘deviant’ cases that do not appear to fit the prevailing pattern or the overall argument ( [40]; p.212) to enhance credibility. Additionally, fair dealing of participants’ views ensured incorporating a range of perspectives which did not favour particular subgroups of the sample [38]. Reflexivity through scrutinising HJB’s positionality and role in the research process [38] was aided by discussions between the authors about the coding and analysis, with the aim of augmenting the analysis rather than label any differences in perspectives as right or wrong. Additional quotes to support the analysis and detailed memos of responses across the sample are also provided for further transparency (Supplementary File 4).

### Questionnaire and anthropometric variables

#### Questionnaire variables

##### Socio-demographic and other individual level variables

Socio-demographic variables included age as a continuous variable from which a dichotomised variable (aged 10–14 years and 15–19 years) was derived, residence type (rural or urban), country of birth, religious and tribal affiliation, ever attended school, current grade, and marital status were all defined as categorical variables. Other individual-level maturation and health variables that may be important covariates in a larger scale study included age at menarche, and number of pregnancies/ children. Only one participant had been married and none reported ever being pregnant and so these variables were not considered further. Health indicators included treatment for malaria infections, which was categorised ranging from currently to never, and for parasitic worms (categorical variable ranging from < 3 months ago to never). The number of available sources of nutrition information was categorised as ‘no access’ (0 sources), ‘limited access’ (1 to 2 sources) and ‘adequate access’ (3 or more sources). The remaining individual level variables were physical activity, sedentary behaviours, and sleep duration. For physical activity, a summary mean score was calculated from the individual items of the PAQ-A (as described above). Each item was scored from 1 (low level of PA) to 5 (high level of PA). Further, the total mean score was categorised as ‘low activity’ (mean score of < 2.75) and ‘adequate activity’ (mean score ≥ 2.76). These cut-off points were adopted based on recommendations from a previous study assessing adolescent physical activity level using PAQ-A [41]. Adequacy of sleep duration was based on criteria defined for paediatric populations [42, 43], as used in other studies [44, 45]. According to these criteria, children aged 10–12 years were classified as having ‘low’ (< 9 h), ‘adequate’ (9 to 12 h) and ‘excess’ (> 12 h) sleep duration. Categories for adolescents aged 13–19 years were ‘low’ (< 8 h), ‘adequate’ (8 to 10 h) and ‘excess’ (> 10 h) sleep duration. For sedentary behaviours, responses were coded 1–5 depending on frequency of occurrence and scores summed.

##### Wider determinants of health (household and community levels)

At the household level, these included family type, living in owned or rented accommodation, parental occupation and education levels, all defined as categorical variables. Community level resources included access to local food markets (standard built markets, supermarkets, shops, open markets (known as *Lumo*) and other types of markets) categorised as ‘little access’ (1 or 2 markets) or ‘adequate access’ (≥ 3 markets). Similarly, access to healthcare services was categorised as ‘not available’ if participants had no access or a traditional midwife in the community, and ‘sufficient access’ for access to a hospital, clinic, and pharmacy. With respect to availability of good roads, no paved road was categorised as ‘unsuitable for vehicles’ (and considered the lowest quality), gravel roads as ‘moderately suitable for vehicles’, and asphalt, basalt or concrete roads as ‘suitable for vehicles’ (and considered the best quality).

#### Anthropometric variables

##### Calculated vs measured height

Estimation of height from ulna length was based on the equation height (cm) = 35.63 + 1.21 × age (years) + 4.06 × ulna (cm) [46]. Height was also estimated as 2 × demi-span length (cm). In the current study, all the participants were able to stand. Estimated height was not needed, and therefore standing height was used to calculate adolescent BMI and underpinned BMI-for-age and height-for-age z-scores.

##### Nutritional status measures

BMI-for-age z-scores, height-for-age z-scores, and waist-hip ratios were calculated from weight, height, and waist and hip circumferences, respectively. In the absence of national nutritional status reference standards available for Gambians, values from existing literature [47–51] were used to derive weight status and stunting categories (see Supplementary File 5).

### Data management and statistical analysis of the questionnaire and measurement data

The questionnaire responses were entered in MS Excel spreadsheets and data cleaning performed to check for accuracy and completeness before being exported to SPSS version 27. Due to the small sample size a 100% check on data entry was performed. Of the 96 questionnaire items per participant, only one item was incorrectly entered (0.03% error rate). After exporting the data to SPSS, data completeness of the attempted questionnaires was assessed to identify the level of missing data and potential problematic questions. The most challenging questions for the participants were the ones that assessed sleep and sedentary lifestyle. Some of the participants, especially in the rural setting, had problems indicating the time they went to sleep or the time it took them to fall asleep, which they attributed to not having a mobile phone. One (3.1%) or two (6.3%) items were missing in each of these variables, overall accounting for 1% of expected data items. The data were therefore imputed from participants’ prayer times. The level of missing data did not reach 10% for any individual variable and was highest (9.4% missing data) for the question related to the type of electricity supply for those who had electricity in their household.

Categorical variables were derived for the individual, household and community-level factors from the questionnaire. Categorical variables were also defined for the nutritional status variables (weight status, abdominal obesity and stunting) using the cut-points as described in Supplementary File 5. Counts and percentages were used to describe the distribution of the variables for the sample overall, and for the urban and rural sub-samples. Pearson’s chi-squared (or Fisher’s exact test when more than 20% of cells had an expected cell count of less than 5) [52] was used for describing differences between rural and urban participants for each categorical variable. The anthropometric continuous variables were examined for normality of the distributions and descriptive statistics used to examine group averages, measures of spread, and differences between rural and urban participants. Mean and standard deviation (SD), and independent samples t-tests were applied to normally distributed data. Non-parametric equivalents – median and interquartile range (IQR), and Mann Whitney U tests – were used for variables which we not normally distributed. Statistical significance for all tests was set at *p* < 0.05. As the study was exploratory, statistical inference of between group or between measure differences in the quantitative analysis should be treated with caution. Further, the intentionally small purposive sample did not support assessing predictors of the outcomes and confounding factors in regression analysis.

## Results

### Data collection sessions

Although the time and date for the data collection session were previously agreed, there was a three hour delay to commencing the sessions in village 1. Participants explained that as girls they can be given additional household or farm chores which they would have to finish and were not permitted to prioritise their research participation. The girls appeared shy in the FGD in this village setting but were engaged by singing at the start of the session to establish rapport and a positive mood, and encouragement of the research team. In village 2, participants were more interactive compared to village 1. The participants were expressive and the mood was light with laughter and jokes. However, because the meeting was conducted in the *Bantaba* at the centre of the community, it was occasionally interrupted by passersby and children playing nearby. The focal person encouraged children not taking part to move away to minimise distraction, but took no other part in the discussions. Seating and tables were available in village 1; however, in village 2 it was necessary to sit on the cement floor on mats to conduct the focus groups. In the questionnaire completion sessions in both rural villages, it was necessary for the research team to support younger participants and those with limited reading skills by reading out the questions and/ or interpreting and explaining them in their preferred language.

It was necessary to conduct both of the urban sessions from late afternoon into the evening (approximately 4–7.30 pm), to accommodate the girls who attended after-school study classes or Quranic school (*Dara*). Both focus group sessions were very interactive and although the focal person was present, they did not get involved in the discussions. The same level of support required in the villages in answering the questionnaire was not needed in the urban setting as most participants were able to fully complete the questionnaire unaided.

### Sample characteristics

In total, 32 female adolescents, with equal numbers from urban and rural settlements participated in the study, therefore the maximum target sample was achieved (Table 2). All participants engaged in the focus group discussions, attempted the questionnaire and completed all of the physical measures. The girls, aged 10 to 19 years (median age 14.5 years), were all born in The Gambia and reported that they were of Muslim faith. Overall, half of the participants identified as being from the Mandinka ethnic group (94% of rural participants). The remainder were from other ethnic groups (including Fula, Wolof, and Jola). All study participants attended school. Although not within the questionnaire, some participants described attending formal English Language taught schools, while others were attending formal Arabic taught schools. However, all those attending Arabic schools were partly taught in the English language. According to age, about 28% of the participants should have been in primary school, however over 60% of the sample were in primary school grades.

**Table 2:** Sample characteristics

Characteristic	N (%)
**Country of birth**	
The Gambia	32 (100)
**Residence**	
Village 1	8 (25.0)
Village 2	8 (25.0)
Urban area	16 (50.0)
**Age**	
10 to 14 years	16 (50.0)
15 to 19 years	16 (50.0)
**Religion**	
Muslim	32 (100)
**Ethnic group**	
Mandinka	16 (50.0)
Other^a^/ Other, not specified	16 (50.0)
**Attends school**	
Yes	32 (100.0)
**School grade**	
1–6 (primary)	20 (62.5)
7–12 (secondary)	12 (37.5)

^a^Other = Fula (9.4%), Wolof (6.3%), Jola (3.1%)

Other, not specified = 31.3%

### Focus group findings

Five themes were identified in the focus group data (Table 3). The five themes were: 1. Cultural norms: harmful vs. beneficial to nutrition related health; 2. Concepts of healthy diet and weight; 3. Approaches to tackling under- and overnutrition; 4. Study recruitment: barriers and facilitators; 5. Study questionnaires and proposed measures are mostly feasible and acceptable. Analysis of the five themes is presented below, supported by direct quotes from the data (additional quotes supporting the analysis can be found in Supplementary File 4). As described in the methods, direct quotes to support the analysis are labelled with a unique reference number, setting (urban or rural) and age. The term ‘parent’ refers to parent or any other guardian.

**Table 3:** Themes and sub-themes from the focus group data

Main themes	Subthemes
1. Cultural norms: harmful *vs.* beneficial to nutrition related health	• Harmful practices • Beneficial practices
2. Concepts of healthy diet and weight	• Rural: high fat and sweetened foods are healthy • Certain food combinations and nutrients are healthy • Overeating leads to obesity • Inactivity leads to obesity • Obesity is natural • Underweight and overweight caused by illness
3. Approaches to tackling under- and overnutrition	• Individual responsibility • Household-level responsibility • Collective action in communities • National solutions
4. Study recruitment: barriers and facilitators	• Parental influence *vs.* young peoples’ autonomy • Focal person important • Peer influence likely • Participation not guaranteed • Building trust • Diverse practical strategies needed
5. Questionnaires and measures are mostly feasible and acceptable	• Questionnaire self-completion: researcher help needed in rural setting • Follow up strategies: remote methods may be challenging • Positive and negative views on blood samples

#### Theme 1. Cultural norms: harmful vs beneficial to nutrition related health

Adolescents in both urban and rural settings identified similar food-related norms and practices that applied in their communities, agreeing that there was a high level of adherence to these practices. As outlined by the participants, these practices included children not being allowed to touch the middle of bowl (where the more prestigious protein foods such as meat and fish are placed) when eating with elders; advised not to eat certain foods such as eggs, bitter tomatoes [a fruit resembling a tomato used as a vegetable in soups and stews], fish heads, or *kew* [clay collected from beaches or river banks and air died; a form of geophagy (the intentional practice of eating soil) observed among Gambian children [53]]; encouraged to eat between lunch and dinner (known as *Sita*); not eating too much; showing respect when eating with elders (i.e. taking small food portions, concentrating on the food, and waiting for elders to serve them meat or fish). Furthermore, it was reported that it was common for men in each household to get the best share of food in terms of quantity and quality. Varied views were also proffered on what they thought were the effects of these practices on their nutrition related health. Discussion about not being permitted to touch the middle of the bowl when eating with elders indicated an instance where participants were negative about an established practice. Across settings there was general agreement that this practice was harmful, sometimes strictly enforced, and could lead to girls’ undernutrition and illness,

*‘If you tell adolescents not to eat meat or fish from the middle of the bowl, it can make them have less weight and can even make them sick’ (P4, rural, 12 years).*

Not being allowed to eat fish heads was not considered relevant for their nutritional status, but not eating eggs, bitter tomatoes, or *kew* was considered beneficial practices that they should adhere to,

*‘Pregnant adolescent girl should not eat dry clay and bitter tomatoes because it will affect the baby when born; They said when a baby is born with rashes that is usually caused by the mother eating bitter tomatoes when pregnant’ (P23, urban, 17 years).*

With respect to men’s share of food, adolescents from the rural area believed that it does not influence their nutritional status. They also agreed that focusing on the food eaten and the practice of *Sita* were beneficial among adolescent girls,

*‘Eating between meals will make you healthy and have good weight’ (P1, rural-13 years).*

#### Theme 2. Concepts of healthy diet and weight

When asked what, in their view, constituted a healthy diet, the rural girls commonly mentioned cooked dishes such as *Domoda* [Groundnut soup], *Benachin* [a one-pot rice, meat and vegetable dish], *Superkanja* [okra soup], and palm oil stew. These are potentially high fat dishes, depending on the proportion of cooking ingredients used such as palm oil in savoury dishes, or were sweetened with sugar,

*‘Eating palm oil stew that has furo [fish], potatoes, bitter tomatoes, cabbage, Kucha [hibiscus leaf] is healthy diet’ (P2, rural, 14 years).*

*‘Healthy diet is composed of mono [porridge] with sugar and sour milk’ (P4, rural, 12 years).*

In the rural areas, but more commonly among the urban adolescents, consumption of specific food groups and nutrients was highlighted,

*‘Is a diet that contain fruits and vegetables’ (P26, urban, 16 years).*

*‘It can be food that contain vitamins and proteins’ (P17, urban, 11 years).*

Adolescents were asked to state their feelings about their own weight. Commonly, the participants in rural and urban settings said they viewed their weight as ‘normal’. However, some considered themselves underweight, and some possibly overweight or obese. Some judged their weight status by indicating how heavy they felt. Others viewed their weight by comparing it with what they considered normal or ideal body weight among the girls in their settings. Those who considered themselves normal weight were mostly satisfied with their weight. Those who deemed themselves underweight or overweight expressed some discontent with their weight status. Each individual adolescent provided an assessment of their weight status, and so comparison of perceived and measured weight status is presented in the quantitative results below.

The adolescents’ views on the causes of undernutrition and overnutrition mostly associated these conditions with the amount of food consumed. However, there were also ideas put forward which contrasted with conventional biomedical views,

*‘If the person did not eat good food like apple, egg, banana and corn they will be underweight’ (P15, rural, 17 years).*

Although not exclusively, girls in the urban setting showed more awareness of scientific views on the causes and prevention of malnutrition than their rural peers. Urban girls mentioned eating a diet low in carbohydrates, proteins, vitamins and not eating the required amount of food can cause undernutrition,

*‘It means the person is not eating food, like foods that will give you energy, like carbohydrates, proteins and vitamins’ (P30, urban, 11 years)*

Across settings, overnutrition was linked to *‘eating too much’ (P6, rural, 13 years)*. In the rural setting this was related to what they deemed as unfavourable eating habits such as variety and eating too regularly. In both settings, eating too much carbohydrate-rich food was considered a cause of overweight, whereas girls in the urban setting were also inclined to highlight energy dense foods as the culprits,

*‘Taking different types of foods like bread, rice and porridge at short intervals can cause overweight or obesity – but if you eat one type of meal, that can reduce the risk of being overweight or obese’ (P8, rural, 14 years)*.

*‘Eating too much oily and sugary foods can cause overweight and obesity’ (P29, urban,16 years).*

Across both settings being inactive was identified as a cause of overnutrition,

*‘If you are eating too much, and you are not exercising your body like doing household chores, can cause overweight and obesity’ (P22, urban, 19 years)*.

Also in the rural villages and the urban area, being overweight was *‘natural’ (P32, urban, 11 years)* or one’s fate for some individuals,

‘*If someone is overweight or obese that is natural sometimes and can also be God making’ (P8, rural, 14 years)*.

#### Theme 3: Approaches to tackling under- and overnutrition

Similar ideas were shared in the rural and urban settings for the management of undernutrition. Suggestions were consistent with views on the causes of undernutrition – eating enough good or ‘healthy’ foods, specific foods or nutrients. They advised that this should be done while observing cleanliness and having rest, including going to bed after eating,

*‘Underweight person should eat garri [cassava porridge], cornflakes and make ponsehwo [bread mixed with sugar and water, occasionally with milk added] at night to increase weight’ (P1, rural, 14 years).*

Suggestions for overweight and obesity treatment were the counterpart of those for underweight; that is, exercising, dieting by reducing the amount of foods/ certain dietary components eaten (such as fats, oils, and sugars), increased consumption of sour foods and drinks, skipping meals, especially dinner, and not going to bed directly after dinner,

‘*They should avoid eating too much and engage in exercise, but sitting at one place is also a sickness on its own’ (P32, urban, 11 years).*

Common across settings was support for medications or food supplements (often originally developed for other purposes) that are promoted in advertising and social media as suitable for addressing under- or overweight,

*‘There are certain drugs when you buy them and drink it you can have weight, like Super apeti, they can be either tablets or syrup’ (P18, urban, 11 years). [Authors note: Super apeti is an over-the- counter medicine containing Cyproheptadine, a 1*^*st*^*-generation antihistamine. Appetite stimulation is a side effect, increasing food intake and inducing weight gain, but it is not recommended for this purpose by the medical profession * [54]*].*

A range of ideas were shared in both settings on household, community, and national level responsibilities and actions for tackling malnutrition, although rural girls were less likely than those in the urban area to voice opinions.

At the household level, many rural adolescents held that parents should provide adequate food to adolescents and this included providing money for adolescents when going to school. Some added that household heads should provide *fish money* [household spending money] for the family, and adolescents should be engaged in family food preparations.

On the other hand, urban girls mentioned the importance of access to loans and parental employment,

*‘Parents should be engaged in paid jobs that will earn them money to be able to cater for their children’ (P22, urban, 19 years)*.

Some rural girls believed parents should help their children reduce eating to control weight. Although not always sure on the appropriate method, some did attempt suggestions,

‘*Parents should advise their children not to eat rice at night’ (P16, rural,16 years).*

Consistent with their discourse on government and community responsibilities (see below), urban girls focused on exercise; proposing that parents should exercise with their children as well as advising them to exercise,

*‘Some adolescents when going to school even a short distance they will take a car. They should be encouraged by their parents to walk to school if the school is not far’ (P22, urban, 19 years).*

Additional suggestions for parents made by the girls in the urban setting included medical check-ups, discouraging eating ‘junk food’, and being aware of their children’s eating behaviours.

In the rural areas it was felt there was collective responsibility within communities for providing food, and in encouraging the eating habits they felt were important in tackling obesity, such as cutting down and skipping meals,

‘*Community members should help adolescent reduce food intake. If they eat in the morning, they should skip the next meal’ (P5, rural, 10 years).*

Girls in the rural villages also contested that community leaders could aid the distribution of help provided by the government to targeted households,

*‘Community leaders should make sure whatever assistance that comes from the government like foodstuffs reached the adolescent girls’ (P9, rural, 17 years).*

Urban girls also focused on community leaders’ roles which they felt should include ensuring free access to exercise facilities, and advocated for increased land accessibility for gardening and animal husbandry. They also emphasised how community leaders could be instrumental in mobilising adolescents, parents, and nutritionists to collectively address adolescent nutrition issues in their respective communities, and provide adolescents with good food and access to medication,

*‘Community leaders should communicate to parents about good nutrition who in turn can advise their children. The chiefs/ Alkalo should also collaborate with researcher and nutrition experts to advise adolescent on good nutrition’ (P31, urban, 10 years)*.

In the urban area, girls tabled various practical ways in which community leaders could effect change, which ranged from organising meetings with parents to discuss adolescent nutrition issues to engaging the government on nutrition strategies beneficial to girls in their communities,

*‘Community leaders conduct meeting with members and put in strategies to engage the government in supporting the community about arising nutritional issues’ (P32, urban, 11 years).*

Several of the adolescents spoke about the significant role they felt government had to play at the national level to manage malnutrition among girls. Some rural girls recommended that the government make several fundamental improvements to community environments,

*‘They should help communities like ours with electricity, good road, standard market and health facility. We walk for long distance to go to the MRC [Medical Research Council field station, Keneba]’ (P16, rural, 16 years).*

In the urban setting, adolescents also proposed the government put in place methods to access nutrition information for both parents and adolescents, and additionally referred to the need for education,

*‘The government should put in strategies to educate parents in the communities about good nutrition, because some parents are not educated and are unaware of good nutrition. In return, they will be able to help their adolescents eat good nutrition’ (P19, urban, 11 years).*

*‘Some people cook unhealthy food in their homes because that’s what they can afford, so the government should provide food for poor households and information for all households either rich or poor’ (P32, urban, 11 years)*.

Additionally, they advocated for access to job opportunities for adolescents after finishing grade 12, free school meals, income subsidy for parents, access to farming and animal rearing facilities, and adequate access to food,

*‘The government should help adolescents with free school meals because the food sold at school is expensive, and sometimes when you buy it you can find a foreign body in it which means you wasted money because you will not eat it. Some food sellers bring leftover foods the following day if that food was not completely sold out the previous day and this can be poisonous to children’ (P24, urban, 15 years).*

The adolescents also indicated the importance, in their view, of providing resources for poor households, as this participant stated,

*‘The government should provide television for some households so that parents are able to watch nutrition programmes like cooking episodes and this can help them give good nutrition advice to their children’ (P30, urban, 11 years).*

Rural girls’ recommendations to address overweight and obesity in their communities included the government providing (or withholding) food assistance, access to nutrition information and medication. In addition to nutrition information and medication access, urban girls recommended that the government ensures that adolescents living with overweight or obesity have free access to a gym, encourages PE in schools, provides access to health facilities for health and nutrition check-ups and medications, and engages parents in adolescent nutrition issues,

*‘Government should provide adolescents with access to free gym without paying’ (P22, urban, 19 years).*

#### Theme 4: Study recruitment: barriers and facilitators

Participants suggested various potential techniques for recruiting respondents to larger studies. Discussion included the possible challenges, enablers as well as motivators for parents and for the adolescents that might be potential future participants.

Across focus groups in both settings the importance of engaging parents in facilitating participant recruitment to the study was highlighted. It was emphasised that the researcher should meet and explain the research relevance to parents, as this quote suggests,

*‘Take time to talk to parents to convince them for their children to participate’ (P4, rural, 12 years).*

Most participants agreed that this method could be implemented by calling parents or conducting house to house visits. Another method suggested by participants from the rural community was for the researcher to organise a meeting with all the community members at the community centre.

Key potential motivating factors for parents highlighted by participants included engaging mothers during the recruitment process, and for the researcher to describe the research in detail to parents. It was considered important that researchers explain the research purpose, procedures for data collection, and the research benefits for their children,

*‘The parents will ask whether the study is beneficial. If they know that it is beneficial for their children then they will let them participate’ (P24, urban, 15 years)*.

In addition, adolescents in the urban setting felt that parents’ awareness about health and nutrition issues and the potential for support to influence children’s health related habits would enhance recruitment,

*‘You know some parents usually advised their children not to eat certain foods but their children do not follow their advice, so when someone outside come to discuss nutritional issues to their children they will be willing to let them participate because they are aware and knows the importance of nutrition and health’ (P19, urban, 11 years).*

The role of parents in recruiting adolescents was important across settings, but views on the extent of parental influence varied between urban and rural settings. Adolescents in the rural villages were inclined to view that once parents agreed their children will also agree to participate without questioning the parental decisions,

*‘If parents agreed to the research, then all participants will agree because it is against our culture to go against your parents’ words’ (P7, rural, 15 years).*

By contrast, participants in the urban setting emphasised young peoples’ autonomy. They maintained that the young people themselves should have adequate and appropriate information about the study, as for their parents, and thus should also be involved in any discussions with their parents. They also suggested that girls ultimately make their own decisions on the issue of participating, and recommended they should be approached with politeness and clarify issues for those in a dilemma about taking part,

*‘You go and discuss with the parents together with the children and if they agree they will participate’ (P 23, urban, 17 years)*

*‘You engage children in the discussion process’ (P22, urban, 19 years)*

Peer influence in general was discussed as an important facilitator of future recruitment in the rural setting, and there was confidence that others would participate. However, it was also proffered that taking part in research in the future may hinge on the experience of those participating in the current study,

*‘After this interview, we will inform the rest of our peers in the community and the benefits in participating and this can encourage them to participate in the main [future] study’ (P15, rural, 17 years).*

*‘Our colleagues will be willing to participate in future study’ (P10, rural, 14 years)*.

*‘Our experience with the study whether good or bad will determine future recruitment’ (P13, rural, 15 years).*

Across the settings, in addition to being made aware of the benefits, practical suggestions such as providing incentives was deemed important for successful recruitment,

*‘You should provide gifts like reading books for adolescents because when they know that they will get something in return for participating, they will be very enthusiastic about it’ (P26, urban, 16 years).*

In terms of other important adults, emphasis was placed on the inclusion of a focal person from the VDC to act as a mobiliser, who in turn could organise meetings with community members to explain the research relevance to members. The role of the *Alkalo* was seen as key to facilitating a range of practical strategies such as house to house visits, visiting schools and madrasas (Islamic schools) within the community,

*‘You will visit the compounds one by one and explain the reasons for the research to individual households and these people will also likely share the information in their neighbourhoods’ (P23, urban, 17 years).*

Participants recommended that these diverse strategies would be successful if appropriate information regarding the research was also shared to all relevant authorities. *Alkalos* were also potentially important for addressing the potential barriers of recruitment of the researchers being strangers in the target communities,

*‘Lot of misunderstanding and delays may happen before some people will accept to participate in the research especially if they don’t recognise you as member of the community. Some may even close their doors to you when you approach them. Is important you talk to the Alkalo to gain access’ (P17, urban, 11 years).*

Linked with the issue of researchers being strangers, building trust by demonstrating connections with the communities and by researchers carrying proof of their credentials were important elements of breaking down barriers,

*‘If you come and talk to parents about the study, they will ask for your identity and some people you know in the community, and if they trust you [the researcher], they can allow their children to participate’ (P22, urban, 19 years)*.

Facilitators of recruitment notwithstanding, the adolescent girls were clear in stating that taking part was not guaranteed if the concerns of adolescents, parents or authorities were viewed to outweigh potential benefits,

*‘Some parents may refuse for their children to take part in the study even after discussing with them’ (P7, rural, 15 years)*.

A key element of the research process that the girls were adamant would result in non-participation in future research would be if injections were part of the data collection,

*‘If the research involves blood and urine collection, people will agree to participate but if it involves injection they will not take part because of issues surrounding COVID-19’ (P13, rural, 15 years).*

#### Theme 5. Survey questionnaires and measures are mostly feasible and acceptable

According to the participants, the questionnaire was not difficult, but in reality, the urban adolescents found completion easier than the rural participants. The urban residents did not mention any specific section or question that was not clear, or was difficult to answer; most of them completed the questionnaire independently and quickly, as previously noted. By contrast, in the rural villages most of the participants were assisted to answer the questionnaire. Rural participants specifically mentioned that the sedentary lifestyle section of the questionnaire was difficult for them to answer. They claimed not having mobile phones made it difficult to report the exact time in relation to questions on going to bed and length of sleep. As noted in the analysis section, reported prayer times were used to estimate the hour they went to bed.

In discussing the feasibility of repeat data collection, including by remote methods such as by phone, most of girls reported not having mobile phones to support direct conversation. This was most common in the rural areas and among the younger adolescent girls. The participants claimed that this would be the case for most of their peers. Alternative suggestions made by the girls included calling participants’ parents, the VDC chair, or participants’ siblings, or for the researcher to buy mobile phones for participants,

*‘You can contact our peers through their parents’ mobiles or the Village Development Committee (VDC) focal person’ (P1, rural, 13 years).*

Specific mobile networks were recommended. Although some stated network quality could accommodate follow up conversations, others were not sure of this because they did not have mobile phones. However, the majority agreed that network quality varies and this can be good or poor depending on the participant’s location at the time of call,

*‘It depends, sometimes the network is very poor and sometimes is good’* (P19, urban, 11 years).

The respondents suggested other avenues to conduct follow up data collection and this included the researcher coming back to the community to meet participants in person, or for someone else to administer the questions on their behalf, calling participants parents or siblings and use of social media for communication. Due to the lack of mobile phone ownership, the researcher coming back to the community was the most recommended approach across all groups,

*‘Most adolescents will not have access to mobile phones, but you can come in person again for follow up if communicating by phone is not possible’ (P30, urban, 11 years).*

Linked with the discourse above relating to factors influencing recruitment, positive and negative attitudes towards giving blood samples were shared by participants,

*‘Some people will be afraid of the needle and that can prevent them from joining’ (P24, urban, 15 years).*

*‘Some adolescents may consider it an opportunity to test their blood and that can encourage them to participate’ (P17, urban, 11 years).*

Adolescents also believed that their willingness to take part in a future larger study would be related to the benefits outlined in Theme 4. However, in the urban group, participants alluded to a fear of surreptitious pregnancy testing, and therefore to the rejection of urine sample collection,

*‘Many adolescents will be more willing to provide blood than urine samples’ (P19, urban, 11 years).*

### Questionnaire and physical measures

#### Questionnaire data

Results for the questionnaire data comparing urban and rural settings are summarised in Supplementary File 6.

##### Individual level factors

Around 75% of the rural sample reported that they had never had malaria; significantly higher than the third of urban participants (*p* = 0.013). Overall, around 66% of participants reported they had never received treatment for parasitic worms, with no statistical difference between settings. Over 50% of the participants reported having started menstruation, with age of menarche at 14 or 15 years old for over 60% of the relevant girls. Only a quarter of the respondents had adequate access to nutrition information (i.e. at least three sources such as school, family, mass media), and all of these girls were in the urban area (*p* = < 0.0001). The overall level of physical activity among girls was low. Commonly across the settings, adequate levels of physical activity in the week preceding the study were only reported by 12 girls. The majority of the participants (81%) were in good health; only six disclosed having ailments (e.g. toothache or headaches) that prevented them from doing exercise, and one of the participants revealed reading as the reason for not engaging in exercise. Most of the participants reported limited sedentary behaviours. More than 60% of respondents did not have access to a mobile phone, and those with access used it for less than two hours a day. Similarly, there was limited access to the internet or television, and those who did were predominantly in rural areas (*p* < 0 = 0.01). Participants in the rural settings were less likely to report adequate average sleep duration compared to their urban counterparts (*p* < 0.001).

##### Household-level factors

The rural participants were significantly more likely than the urban girls to live in extended rather than nuclear families (*p* = 0.009). Large families were common and nineteen (59%) of the girls had five or more siblings. Parents or grandparents owned the home they lived in for most of the sample. Around half of the adolescents stated their parents had no formal education; however, a father with no education was more commonly reported in the rural setting. Mothers and fathers were most likely to be in paid employment, but both also did part-time or caring work, and a small number of parents were reported as deceased. Tap water was the source of drinking water for most of the participants. More than 90% of the rural inhabitants shopped for their daily food necessities in their communities. The majority of the urban adolescents, but only a third of those in the rural villages had a source of electricity in their homes (*p* = 0.004). Among the respondents who had electricity in their houses, the national provider, National Water and Electricity Company (NaWEC), was the source for 75% of the urban participants (and none of the rural girls), with solar power or a generator being the other sources.

##### Community-level factors

There were significant differences (*p* < 0.0001) between urban and rural participants in accessibility of food markets. Half of the respondents from the urban area reported access to a variety of built markets such as supermarkets which was not the case for rural girls. Similar variations for resources such as healthcare services, roads, electricity, and schools were observed. Access to biomedical or conventional healthcare such as hospitals, clinics and pharmacies was only available in the urban area. By contrast two thirds of rural respondents stated access to only traditional healthcare, with nearly 40% having no access to any form of healthcare services in their community. Similarly, urban females reported access to good quality (asphalt and concrete) roads, whereby in the rural communities there was only access to paved gravel or unpaved roads. Consistent with reports for household electricity supply, NaWEC electricity was available in the urban areas only, while the rural communities relied on privately installed solar energy (*p* < 0.0001). Almost all the participants attended school. Only a small number (*n* = 12) had to travel outside their communities to attend school; however, for the rural girls this was due to a lack of available schooling in their grade, compared to the urban girls where this was to do with other reasons such as attending a better school (*p* = 0.001).

#### Modifications to the questionnaire

A small number of minor modifications to the questionnaire were identified during the questionnaire sessions (see annotations on the questionnaire in Supplementary File 3), suggesting high face and content validity. Changes included adding the type of school attended (English taught or Arabic school); traveling to a neighbouring town as a source of drinking water; increased choice of roles and mother/ father has died as additional response options for parents’ occupation questions; street food vendor added to the response options for food markets available in communities; generator as a source of electricity supply in the household or community; and liking the school as an option for the reason for travelling to attend school. Urban–rural differences in being able to complete the questions on sleep patterns (noted above) were largely due to diverse literacy ability, with particular difficulty among the rural respondents. The research team decided not to change or omit the questions due to their comparability with existing work [43]. However, the need for the researchers to support participants in sleeping or waking hours using prayer, certain meals, and television news times, daylight or darkness was important to note for future research phases.

The focus group discussions also informed the addition of two new sections of questions for future use of the questionnaire (see Supplementary File 3). One section included indicators of nutrition awareness motivated by the discussions around girls’ own concepts of healthy eating and weight in which they cited certain dishes, foods, or nutrients. Additional questions included matching of nutrients with food groups that are key sources of those nutrients in the diet, and providing a definition of a balanced diet by selecting from a choice of options. The other section included additional questions to ascertain the extent of the nutrition-related cultural practices described in the FGDs, and obtain views on the potential influence of cultural practices on the quantity and quality of food intake.

#### Physical measures

Predicted height (from ulna length and demi-span) and measured heights were significantly correlated with each other (*r* = 0.78–0.85; *p*-value < 0.001). Although predicted height was not needed, as all participants could stand, it can be used as an alternative to measured height in future studies in the Gambian setting where needed. All other anthropometric physical measures by setting are summarised in Table 4. Median weight, height, mid upper arm circumference (MUAC), waist and hip circumferences, and waist: hip ratios were similar across settings. There was a high correlation between BMI and MUAC (*r* = 0.775; *p* < 0.001). BMI for age z-scores were lower among rural compared to urban participants (*p* = 0.017). Average height for age z-scores were also lower among rural than urban girls, however this was not statistically significant.

**Table 4:** Anthropometric measures, by setting

Anthropometric measures							
	**Mean (SD)**			**Median (Interquartile range)**			
	**All**	**Urban**	**Rural**	**All**	**Urban**	**Rural**	*P*- Value*
Weight, kg	45.35 (10.71)	47.25 (12.98)	43.44 (7.81)	45.60 (36.78, 51.33)	48.05 (35.80, 57.63)	45.30 (37.48, 48.98)	0.381
Height, cm	154.85 (11.74)	155.32 (13.60)	154.38 (9.96)	157.00 (149.50, 161.75)	157.00 (147.25, 164.55)	156.80 (150.03, 160.75)	0.780
Mid upper arm circumference, cm	24.69 (9.88)	26.95 (13.67)	22.42 (2.00)	23.05 (21.00, 25.38)	24.30 (20.85, 26.30)	22.25 (21.05, 23.78)	0.138
Waist circumference, cm	65.01 (6.60)	65.90 (8.210	64.12 (4.58)	64.55 (60.50, 69.80)	65.70 (59.93, 73.73)	64.50 (60.88, 67.50)	0.669
Hip circumference, cm	84.00 (9.72)	85.41 (11.30)	82.59 (7.96)	83.50 (77.18, 92.58)	86.65 (77.16, 96.05)	83.50 (76.50, 87.50)	0.445
Waist-Hip ratio, cm	0.77 (0.04)	0.77 (0.05)	0.77 (0.04)	0.77 (0.75, 0.82)	0.77 (0.75, 0.82)	0.77 (0.76, 0.82)	0.809
BMI for age z-scores	−1.03 (0.97)	−0.62 (1.09)	−1.44 (0.63)	−1.00 (−2.00, −1.00)	−1.00 (−1.00, 0.00)	−1.50 (−2.00, −1.50)	0.017*
Height for age z-scores	−1.00 (1.14)	−0.63 (1.31)	−1.38 (0.81)	−1.00 (−2.00, 0.00)	−0.50 (−2.00, 0.00)	−1.00 (−2.00, −1.00)	0.140

^*^For differences between urban and rural samples, significant *p*-value (*p* < 0.05) is in bold type

#### Nutritional status and weight perception

Overall, a similar proportion of participants were classified as underweight or normal weight based on BMI-for-age z-score cut-points (31%; 59%, respectively) or MUAC cut-points (28%; 56%) (Table 5); however, significantly more participants were underweight in rural vs urban settings, according to BMI-for-age z-score cut-points (50% vs 12.5%, *p* = 0.03; data not shown in the table due to small numbers in categories). Around 10% (BMI z-scores) or 16% (MUAC) were classified as living with overweight or obesity according to these two measures. Based on measures of abdominal obesity 21% or 31% of the girls could be considered as having excess abdominal fat according to their waist: hip ratios or waist circumference measures, respectively. Based on height for age z-score cut-points, 41% (*n* = 13) of the girls experienced stunting.

**Table 5:** Nutritional status measures and weight status perception

Nutritional status indicator	N (%)*
	All
Weight status (body mass index for age z-scores):	
Underweight	10 (31.3)
Normal weight	19 (59.4)
Weight status (mid upper arm circumference age-specific cut-points):	
Underweight	9 (28.1)
Normal weight	18 (56.3)
Overweight/ obese	5 (15.6)
Abdominal obesity (waist: hip ratio):	
Excess abdominal fat	10 (31.3)
Abdominal obesity (waist circumference):	
Excess abdominal fat	7 (21.9)
Stunting (height for age z-scores):	
Stunted	13 (40.6)
**Measured (body mass index for age z-scores) vs perceived weight status**	
Underweight perceived as underweight	6 (24.0)
Underweight perceived as normal weight/ overweight	19 (76.0)

^*^Where counts were ≥ 4

Overall, over 70% of participants classed as normal weight when measured also perceived themselves as such, and all girls classed as overweight or obese by BMI z-scores perceived themselves as being of normal weight, when asked during the focus groups. However, 70% of those classified as underweight also perceived their weight as normal (Table 5). A similar picture was found with weight status assigned by MUAC categories, except that some of the girls deemed overweight perceived themselves as underweight.

## Discussion

The purpose of this study was to conduct formative research to understand cultural contexts relevant to nutritional status, feasibility and appropriateness of recruitment methods, self-reported questionnaire and anthropometric measures, views on providing biological samples in the future, and plausibility of data collected among adolescent girls living in urban and rural Gambia. Participants disclosed common cultural practices, understanding of which may be vital in documenting and addressing malnutrition among female adolescents. Further, traditional beliefs and myths rather than conventional biomedical knowledge were more prominent in views on healthy eating and the links between nutrition and body weight among rural participants compared to their urban counterparts. Discussion around perceptions of healthy diet and weight disclosed low levels of awareness of nutrition, which was particularly apparent among the rural girls. This learning will inform assessing the extent and importance of these practices and of nutrition knowledge in a larger scale study. However it also signals the importance of valuing different forms of knowledge where possible [55], and the potential impact of access to good quality education as a determinant of health that spans individual to national levels of influence [56]. Participants suggested various strategies such as involving relevant gatekeepers, a range of study awareness raising activities such as group meetings, house to house visits, involving young people throughout, and the importance of incentives (such as books and stationery) for engaging them in research. The adolescent girls also shared some challenges such as refusal to participate linked to fear of injections and distrust of research. It was also important to have an understanding of the girls’ day to day pattern of activities, such as attending school and supplementary schooling, and household chores and how these impact on taking part. Collectively, these insights were highly informative for enhancing engagement with larger-scale studies.

Good quality questionnaire and anthropometric data were obtained in this exploratory study, evidenced by negligible missing data and the plausibility of the findings. The modifications outlined will further enhance the performance of the questionnaire. Urban–rural differences in the potential household and community predictors of malnutrition (such as access to electricity and good quality roads) was consistent with the picture seen in national data [57, 58]. Nutritional status outcome data obtained showed consistency between estimates of underweight between the measures, and in comparison with previous research among older adolescents [1, 2, 4]. Estimates of overweight were more variable but supports the use of multiple measures to mitigate against inherent limitations. For example, a large waist could be due to fluid retention and stomach distention associated with malnutrition or illnesses such as parasitic worm infection [59]. The findings are further integrated into the wider literature below.

### Cultural contexts and views on diet and weight

The narratives in the current study indicated that the traditional way of communal eating, and practices such as males in the household having priority over protein-rich foods, remain common in both rural and urban households [60, 61]. Qualitative exploration of cultural food practices has previously been carried out in The Gambia [3, 62, 63] and other West African countries [64]. Much of this research has focused on rural women’s views of these practices and how they influence feeding their young children [62–64]. The negative impact of food taboos on maternal and child feeding are emphasised, although Mwangome et al. [63] also highlight cultural mores that have positive impact on hygiene and childcare, and the intersection between culture and wider determinants such as poverty. To date there has been limited inclusion of the views of adolescents on cultural food practices and the influence on nutrition [3]. Analysis of national data examining the determinants of child nutritional status [65] or of healthy eating [66] in The Gambia have no [65] or limited [66] consideration of factors beyond the individual level. Future research would benefit from inclusion of household and community cultural norms and practices in elucidating determinants of and solutions to adolescent malnutrition.

There was high agreement between perceived and measured weight status. However, there was a suggestion that both underweight (potentially due to its high prevalence) and overweight may be normalised. Misperception of weight status among adolescents is common across diverse populations and settings [67–71] and can impact on health as correct weight perception contributes to appropriate weight control habits [68]. Research from Africa on this topic is limited, but a study among adolescents in the Seychelles suggests underestimation of overweight and obesity is associated with a positive view of large body size [68]. Traditionally, overweight is viewed as healthier than normal weight, and a sign of beauty (particularly among women), wealth, success and prestige in The Gambia [72]. This may be a contributory factor in rising obesity levels particularly among the more affluent [73].

Urban girls in the current study were encouraged by their parents to walk to school. The parent–child relationship and parental engagement in activities, as important individual-household level interactions, encourages young peoples’ participation in exercise [74, 75]. More widely, studies conducted in a range of settings, including several sub-Saharan African countries, indicate parental involvement has a beneficial role in healthy BMI and diet [76], adequate physical activity levels [77], decreased engagement in risky behaviours (such as substance, tobacco, and alcohol use) [78], and lower likelihood of poor mental health [79]. Nonetheless, parental involvement in adolescents’ lives may need more support in families of low socioeconomic circumstances and education levels compared to their counterparts with better access to resources [80].

Participants’ own perspectives on addressing under- and overweight support consideration of social determinants and inform strategies for action beyond observational research (and therefore across levels of influence from a SEM perspective [6]). Some of their recommendations (such as the provision of school meals, nutritional education for school children and members of the public, and government support for families to supplement household food consumption) are measures promoted by UNICEF to reduce childhood malnutrition [81] and should be supported as acceptable interventions for young females. These and other ideas (including small-scale horticulture) have provided formative intelligence for research developing a new MRC-funded community-based intervention for improving malnutrition for adolescents (reference: APP30152), led by the authors and currently underway. Urban agriculture has been shown to improve household income, food security and dietary diversity [82]; however, challenges such as water shortages can impact on the success of such programmes [83]. The importance of public contributors’ views in shaping the new study will be fed back to communities as part of wider dissemination of the current research. There was little mention among the study participants of nutrient supplementation or food fortification. This may suggest a significant gap in the health education received at school, is consistent with reported limited access to nutrition information resources among this age-group in Nigeria [84], and should be rectified in ongoing actions to address malnutrition.

### Perspectives on research approaches

The adolescents stressed the importance of the role of parents in potentially engaging in a research study. Participants clearly value parental input, and are also able to see the benefits for parents of their child’s participation (such as reinforcing parental guidance on healthy eating). Among the urban girls this view was balanced with emphasis on their own autonomy particularly the need to be fully informed, and the right to decide not to participate even if their parents agree. Parental support for autonomy is associated with a number of positive outcomes in childhood and adolescence [85]. However, the degree of child autonomy varies by geographical location and thus cultures, and evidence suggests that some parental behaviours that support autonomy (e.g. offering choices) might be less relevant in collectivist cultures than other behaviours (e.g. perceiving information from a child’s point of view) [86]. Empowering women, children and adolescents is deemed essential to address childhood malnutrition in The Gambia [87], and globally stalled maternal and child mortality [88], requiring culturally sensitive approaches [89]. Urban–rural differences in views in The Gambia indicate that cultural and structural nuances in adolescent autonomy within collectivist societies need to be further understood.

Informed consent and willingness to participate is thus influenced by the young people themselves and their parents, but also other influential members of the community, such as *Alkalos*, as the findings suggest. Traditional leaders in low income countries exercise considerable power and influence and generally enhance taking part in research when they are involved [90]. As essentially quasi-government officials, they have a vested interest in the health and wellbeing of their communities, maintain societies traditional social values, and tend to be at the forefront of long-established, extensive, dissemination networks [91, 92]. Further, with access to resources such as mobile phones, they can be important gatekeepers of participant retention and long-term follow-up. However, such influence does raise important ethical issues for engaging young people in research. There is international consensus that child autonomy overrides parental or other adults’ consent (https://childethics.com/charter/) although, as noted above, this may be practiced differentially by setting in the current study. It was important in this and future phases to be mindful of the potential for children to feel coerced into taking part, in particular by being alert to indirect verbal and non-verbal dissent (https://childethics.com/charter/).

Our data supports the contention that adolescents are often enthusiastic about research; however, when biological samples are involved this can impact on willingness to participate [93]. A number of research studies involving biological samples have been carried out in The Gambia [94–96], and negativity about giving blood samples has been previously explored [96]. Concerns included viewing samples as a loss of blood and therefore strength, which would be particularly harmful when harvesting crops if this coincided with study participation [96]. There is little empirical evidence on children’s views on discomfort during research, including needle-related procedures. Qualitative exploration of experiences of children involved in clinical research reports some participants feeling that the needle-related procedure was the worst part of the study because of the pain it caused, and some refused such procedures because of previous painful venepuncture experiences [97]. Quantitative comparison among 8–18 year olds indicated skin prick tests to cause less discomfort, and venepuncture around the same discomfort as a dental check-up [98]. Young people themselves have recommended distraction, providing age-appropriate information, and reducing the duration of lengthy procedures [97, 98] to overcome the barriers to providing samples, and other authors suggest incentives [93, 99]. Given that previous research on children’s views has largely focused on blood samples and not urine samples, and has been conducted in high income countries [97, 98], it is an area of research that warrants further exploration in LMIC settings.

### Strengths and limitations

This study contributes novel data exploring the potential for extending research on nutrition-related health among adolescents, providing a foundation for furthering the evidence-base on this topic in The Gambia. The study confirmed the importance of community focal persons in engaging young people in research in The Gambia. Additionally, we gained nuanced understanding of time and other resources required, particularly around the need to engage with a number of gatekeepers due to wariness, and large geographical spaces in the urban setting. Respondents were recruited in contrasting regions of the country supporting urban–rural comparisons regarding cultural contexts, feasibility of questionnaire completion and of the physical measurements. Plausibility and trustworthiness of the data was indicated through rich narratives shared in the focus groups [100], physical measurements which avoided the under- or overestimation of values associated with self-reported measures [101], and the congruence between the data from the physical measures and what is currently known on the double burden of malnutrition in The Gambia [1, 3, 4]. The questionnaire did not include questions on dietary intake, as this had previously been conducted among adolescents by the first author [4]. Detailed prospective dietary assessment was carried out in a later phase of the research, to be reported in a forthcoming publication. FGDs provide access to group norms and language through group interactions, and are time efficient compared to individual interviews [102]. However, there are potential challenges associated with this method of data collection. These might include dominating or reticent participants, and overtalking among speakers. Nevertheless, these issues were managed by the lead author and fieldworkers, skilled in facilitating and supporting FGD and all fluent in the preferred languages of the participants [102]. With the focus on girls’ own perspectives, gender norms inherently shaped girls’ views, and were also evident in the nuances of the research experience such as the delay to data collection sessions due to gendered roles such as house or farm work. However, not asking specific questions to obtain opinions on how experiences and norms impacted girls differently to boys is a study limitation. The potential for recall bias associated with the questionnaire measures is inherent, particularly among the younger participants who could find it difficult to recall retrospective events [103]. Further, the small sample size and convenience sampling technique, with the aid of the gatekeepers to recruit appropriate respondents, are limitations which could lead to selection bias [104]. However, the essential importance of engagement with community leaders was confirmed in the qualitative data and the experience of conducting the study. Supporting participants to understand some questionnaire questions was required. This has resource implications in terms of having sufficient personnel to support younger and less literate participants in a larger scale study with these target group and settings. By design, this cross-sectional study was small in scale in order to facilitate in-depth exploration. As such, statistical inference of between group differences in the quantitative analysis should be treated with caution. Further, as the aim of the study was not to obtain statistically representative data this does place limits on generalisability of the findings. However, the objectives of the study were met and it is likely that the findings resonate with other adolescents in urban and rural settings in Gambia (and potentially similar settings in other West African countries) and for researchers who wish to work with them. Causal inference between potential predictors and nutritional status outcomes was beyond the scope of the current project.

## Conclusion

There is limited existing research in The Gambia which considers the wider determinants of malnutrition among adolescent girls. The aim of this exploratory study was to conduct formative research in urban and rural areas to understand cultural contexts relevant to nutritional status, feasibility and appropriateness of recruitment methods, self-reported questionnaire and anthropometric measures, and views on providing biological samples. With some enhancements to the research approach and data collection tools, the findings suggest acceptability and feasibility of a larger scale studies of this nature among the target group. Plausible data were obtained which reflect the existence of a double burden of malnutrition in The Gambia, and potentially important individual, household and community influences on malnutrition were identified. The role of empowering autonomy, harnessing agency, and centring their views warrants further attention in improving nutrition-related health among adolescent girls. Urban–rural variance in influences and outcomes, enablers of and challenges to participating in research are also key considerations for future research in this population group.

## Supplementary Information

Supplementary Material 1. A. COREQ checklist for qualitative studies and B. STROBE checklist for cross-sectional studies

Supplementary Material 2. Interview guide

Supplementary Material 3. Questionnaire

Supplementary Material 4. A. Additional quotes supporting the qualitative analysis and B. Focus group memos

Supplementary Material 5. Cut points for nutritional status categories

Supplementary Material 6. Questionnaire results

## Data Availability

All data generated or analysed during this study are included in this published article [and its supplementary information files].
