# Supplementary material for: Factors influencing malnutrition among adolescent girls in The Gambia: a mixed-methods exploratory study
Source: BMC Public Health. 2025 Jan 8;25:80. doi: 10.1186/s12889-024-21242-w (PMC11708179; doi:10.1186/s12889-024-21242-w)
Supplement: Supplementary file 1 — Supplementary Material 1. A. COREQ checklist for qualitative studies and B. STROBE checklist for cross-sectional studies [file 12889_2024_21242_MOESM1_ESM.docx]

**A. COREQ checklist for qualitative studies [qualitative study elements]**

| **Item** | **Guide questions/description** | **Reported on**  **Page no (for peer review stage)** |
| --- | --- | --- |
| **Domain 1: Research team and reﬂexivity** |  |  |
| ***Personal Characteristics*** | | |
| 1. Interviewer/facilitator | Which author/s conducted the interviews or focus groups? | HJB; 14 |
| 2. Credentials;  3. Occupation;  4. Gender;  5. Experience and training | What were the researcher’s credentials? E.g. PhD, MD  What was their occupation at the time of the study?  Were the researchers male or female?  What experience or training did the researchers have? | 14 |
| ***Relationship with participants*** | | |
| 6. Relationship established;  7. Participant knowledge of the interviewer | Was a relationship established prior to study commencement?  What did the participants know about the researcher? e.g. personal goals, reasons for doing the research | 15 |
| 8. Interviewer characteristics | What characteristics were reported about the interviewer/facilitator? e.g. Bias, assumptions, reasons and interests in the research topic | As reported for items 1-5 |
| **Domain 2: study design** |  |  |
| ***Theoretical framework*** | | |
| 9. Methodological orientation and Theory | What methodological orientation was stated to underpin the study? e.g. grounded theory, discourse analysis, ethnography, phenomenology, content analysis | 18 |
| ***Participant selection*** | | |
| 10. Sampling | How were participants selected? e.g. purposive, convenience, consecutive, snowball | 8-9; Table 1 |
| 11. Method of approach | How were participants approached? e.g. face-to-face, telephone, mail, email | 9-11 |
| 12. Sample size | How many participants were in the study? | 2, 23 |
| 13. Non-participation | How many people refused to participate or dropped out? Reasons? | Not known |
| ***Setting*** | | |
| 14. Setting of data collection | Where was the data collected? e.g. home, clinic, workplace | 15-17 |
| 15. Presence of non-participants | Was anyone else present besides the participants and researchers? | 17 |
| 16. Description of sample | What are the important characteristics of the sample? e.g. demographic data, date | 23-24; Table 2 |
| ***Data collection*** | | |
| 17. Interview guide | Were questions, prompts, guides provided by the authors?  Was it pilot tested? | 11-12; Supplementary file 2 |
| 18. Repeat interviews | Were repeat inter views carried out? If yes, how many? | No |
| 19. Audio/visual recording  20. Field notes  21. Duration | Did the research use audio or visual recording to collect the data?  Were ﬁeld notes made during and/ or after the interview or focus group?  What was the duration of the interviews or focus groups? | 15-17 |
| 22. Data saturation | Was data saturation discussed? | 9,18 |
| 23. Transcripts returned | Were transcripts returned to participants for comment and/or correction? | No |
| **Domain 3: analysis and ﬁndings** |  |  |
| ***Data analysis*** | | |
| 24. Number of data coders | How many data coders coded the data? | One |
| 25. Description of the coding tree | Did authors provide a description of the coding tree? | 24-25; Table 3 |
| 26. Derivation of themes | Were themes identiﬁed in advance or derived from the data? | 18 |
| 27. Software | What software, if applicable, was used to manage the data? | N/A |
| 28. Participant checking | Did participants provide feedback on the ﬁndings? | No |
| ***Reporting*** | | |
| 29. Quotations presented | Were participant quotations presented to illustrate the themes/ﬁndings? Was each quotation identiﬁed? e.g. participant number | 25-39 |
| 30. Data and ﬁndings consistent | Was there consistency between the data presented and the ﬁndings? | 25-39 |
| 31. Clarity of major themes | Were major themes clearly presented in the ﬁndings? | 25-39 |
| 32. Clarity of minor themes | Is there a description of diverse cases or discussion of minor themes? | 25-39 |

From: Tong A, Sainsbury P, Craig J. Consolidated criteria for reporting qualitative research (COREQ): a 32-item checklist for interviews and focus groups. International Journal for Quality in Health Care. 2007;19(6):349-57

**B. STROBE checklist for cross-sectional studies [quantitative study elements]**

|  | Item No | Recommendation | Reported on  Page number (for peer review stage) |
| --- | --- | --- | --- |
| **Title and abstract** | 1 | (*a*) Indicate the study’s design with a commonly used term in the title or the abstract | 1,2 |
|  |  | (*b*) Provide in the abstract an informative and balanced summary of what was done and what was found | 2-3 |
| Introduction | | |  |
| Background/rationale | 2 | Explain the scientific background and rationale for the investigation being reported | 3-5 |
| Objectives | 3 | State specific objectives, including any prespecified hypotheses | 5-6 |
| Methods | | |  |
| Study design | 4 | Present key elements of study design early in the paper | 6 |
| Setting | 5 | Describe the setting, locations, and relevant dates, including periods of recruitment, exposure, follow-up, and data collection | 7-8,10-11 |
| Participants | 6 | *Cross-sectional study*—Give the eligibility criteria, and the sources and methods of selection of participants | 8-9 |
| Variables | 7 | Clearly define all outcomes, exposures, predictors, potential confounders, and effect modifiers. Give diagnostic criteria, if applicable | 19-21 |
| Data sources/ measurement | 8* | For each variable of interest, give sources of data and details of methods of assessment (measurement). Describe comparability of assessment methods if there is more than one group | 19-21; Supplementary Files 3 and 5 |
| Bias | 9 | Describe any efforts to address potential sources of bias | 12-13 (questionnaire validity); 19-21 (data collection protocols); 12-15 (data collection training) |
| Study size | 10 | Explain how the study size was arrived at | 9 |
| Quantitative variables | 11 | Explain how quantitative variables were handled in the analyses. If applicable, describe which groupings were chosen and why | 19-23 |
| Statistical methods | 12 | (*a*) Describe all statistical methods, including those used to control for confounding | 22-23 |
|  |  | (*b*) Describe any methods used to examine subgroups and interactions | 22-23 |
|  |  | (*c*) Explain how missing data were addressed | 22 |
|  |  | (*d*) *Cross-sectional study*—If applicable, describe analytical methods taking account of sampling strategy | Not applicable |
|  |  | (*e*) Describe any sensitivity analyses | Not applicable |

| Results | | |  |
| --- | --- | --- | --- |
| Participants | 13* | (a) Report numbers of individuals at each stage of study—eg numbers potentially eligible, examined for eligibility, confirmed eligible, included in the study, completing follow-up, and analysed | 7-8 |
|  |  | (b) Give reasons for non-participation at each stage | Not applicable |
|  |  | (c) Consider use of a flow diagram | Not applicable |
| Descriptive data | 14* | (a) Give characteristics of study participants (eg demographic, clinical, social) and information on exposures and potential confounders | 23-24 |
|  |  | (b) Indicate number of participants with missing data for each variable of interest | 22 |
| Outcome data | 15* | *Cross-sectional study—*Report numbers of outcome events or summary measures | 40-45; supplementary File 6 |
| Main results | 16 | (*a*) Give unadjusted estimates and, if applicable, confounder-adjusted estimates and their precision (eg, 95% confidence interval). Make clear which confounders were adjusted for and why they were included | Not applicable |
|  |  | (*b*) Report category boundaries when continuous variables were categorized | 19-21 |
|  |  | (*c*) If relevant, consider translating estimates of relative risk into absolute risk for a meaningful time period | Not applicable |
| Other analyses | 17 | Report other analyses done—eg analyses of subgroups and interactions, and sensitivity analyses | Not applicable |
| Discussion | | |  |
| Key results | 18 | Summarise key results with reference to study objectives | 45-46 |
| Limitations | 19 | Discuss limitations of the study, taking into account sources of potential bias or imprecision. Discuss both direction and magnitude of any potential bias | 51-52 |
| Interpretation | 20 | Give a cautious overall interpretation of results considering objectives, limitations, multiplicity of analyses, results from similar studies, and other relevant evidence | 44-49 |
| Generalisability | 21 | Discuss the generalisability (external validity) of the study results | Not applicable |
| Other information | | |  |
| Funding | 22 | Give the source of funding and the role of the funders for the present study and, if applicable, for the original study on which the present article is based | 53 |

From: von Elm E, Altman DG, Egger M, Pocock SJ, Gotzsche PC, Vandenbroucke JP, et al. The Strengthening the Reporting of Observational Studies in Epidemiology (STROBE) statement: guidelines for reporting observational studies. Lancet. 2007;370(9596):1453-7
