# Supplementary material for: Factors influencing malnutrition among adolescent girls in The Gambia: a mixed-methods exploratory study
Source: BMC Public Health. 2025 Jan 8;25:80. doi: 10.1186/s12889-024-21242-w (PMC11708179; doi:10.1186/s12889-024-21242-w)
Supplement: Supplementary file 2 — Supplementary Material 2. Interview guide [file 12889_2024_21242_MOESM2_ESM.docx]

**Focus group interview guide**

| **Cultural norms and practices** |
| --- |
| 1. What do you think are the cultural practices associated with female adolescent nutrition? |
| 1. How do you think they influence their nutrition? |
| 1. Of those cultural practices, which do you think might be beneficial to adolescent girls' nutritional status? |
| Why do you think they are beneficial? |
| 1. Of those cultural practices, which do you think might be harmful to adolescent girls' nutritional status? |
| Why do you think they are harmful? |
|  |
| **Views on diet, underweight, overweight** |
| 1. What do you think comprises a healthy diet? |
| 1. Please tell me what you think of your weight? |
| 1. If someone was underweight, what would you think were the reasons for that? |
| 1. How do you think underweight can be prevented? |
| 1. If someone was overweight or obese, what would you think were the reasons for that? |
| 1. How do you think overweight and obesity can be prevented? |
|  |
| **Views on addressing adolescent malnutrition** |
| 1. How do you think undernutrition among adolescent girls can be addressed at the national level? |
| 1. How do you think undernutrition among adolescent girls can be addressed at the community level? |
| 1. How do you think undernutrition among adolescent girls can be addressed at the household level? |
| 1. How do you think overnutrition among adolescent girls can be addressed at the national level? |
| 1. How do you think overnutrition among adolescent girls can be addressed at the community level? |
| 1. How do you think overnutrition among adolescent girls can be addressed at the household level? |
|  |
| **Engaging adolescents in research** |
| 1. What method do you think is most appropriate to engage adolescent girls in research projects where you live? |
| 1. How can this method be implemented? |
| 1. What other methods do you know of/ do you think might work? |
| 1. What is the best way to reach people in your communities? |
| 1. What are the problems that you think I may encounter in recruiting participants? |
| 1. Can you tell me the things that you think will encourage parents and guardians to allow their children to participate in a study? |
| 1. Please tell me about the things you feel would particularly attract adolescent girls into taking part in a study. |
| 1. How willing do you think participants would be to provide blood samples in a research study? |
| 1. How willing do you think participants would be to provide a urine sample in a research study? |
| 1. What access do you have to mobile phones? |
| 1. Which network works best here? |
| 1. Can the network quality accommodate clear and long mobile conversation? |
| 1. What other alternative to phone contact do you think would be feasible if we wanted to meet with participants more than once? |
|  |
| **Completing the questionnaire** |
| 1. How did you find the questionnaire to complete? |
| 1. Please tell me about any of the questions that were difficult to understand or answer. |
|  |
