# Supplementary material for: Factors influencing malnutrition among adolescent girls in The Gambia: a mixed-methods exploratory study
Source: BMC Public Health. 2025 Jan 8;25:80. doi: 10.1186/s12889-024-21242-w (PMC11708179; doi:10.1186/s12889-024-21242-w)
Supplement: Supplementary file 3 — Supplementary Material 3. Questionnaire [file 12889_2024_21242_MOESM3_ESM.pdf]

QUESTIONNAIRE

Section 1: About you

1. How old are you? Age (in years): ✐

2. What is your country of birth? Country name: ✐

3. What is your religion?

A. Muslim [ ]	B. Christian [ ]	C. Other, please specify: ✐

4. What is your tribe?

A. Mandinka [ ]	B. Fula [ ]	C. Wolof [ ]
D. Jola [ ]	E. Other, please specify: ✐	

5. Have you ever been to school?

A. Yes [ ]	B. No [ ]

If **'Yes'**, answer Questions 6 and 7. If **'No'** skip to Question 8

6. What type of school are you attending?*

A. English [ ]	B. Arabic [ ]

7. What grade are you in currently?

Dropped out [ ]	B. Grade 1-6 [ ]	C. Grade 7-9 [ ]	D. Grade 10-12 [ ]

8. How many brothers and sisters do you have? Write 0 if you don't have any Number: ✐

9. What type of family do you live in?

A. Nuclear family (father, mother, sister/s and brother/s)	[ ]
B. Extended family (father, mother, sister/s, brother/s, other family members)	[ ]
C. Single parent family (one parent: a mother or father)	[ ]

Factors influencing malnutrition among adolescent girls in The Gambia: A mixed-methods pilot study. *Text in red denote modifications to the questionnaire based on study findings.

D. Step or blended family (two separate families coming together – for example a new husband or wife and children from previous marriages coming together) [ ]	
E. Grandparent family (parents of mother or father) [ ]	
F. Other family type not listed, please specify:	✐

10. Do you live in a rented home (e.g. apartment or house)

A. Yes [ ]	B. No [ ]

If '**No**' answer Question 10. If **'Yes'** skip to Section 2.

11. Who owns the home (e.g. the apartment or house) you currently live in? Tick **all** that apply if more than one owner.

A. My father	[ ]
B. My mother	[ ]
C. My grandparents	[ ]
D. Another family member	[ ]
E. A family friend	[ ]
F. Other family type not listed, please specify:	✐

Section 2: Your health and social circumstances

12. How old were you when you started menstruating? Age (in years): ✐

13. When was the last time you saw your menses?

A. I am currently seeing my menses	[ ]
B. Less than a month ago	[ ]
C. A month ago	[ ]
D. More than a month ago	[ ]

14. Have you ever been married?

A. Yes [ ]	B. No [ ]

If **'Yes'** answer Questions 15 and 16. If **'No'** skip to Question 17.

15. Are you currently married?

A. Yes [ ]	B. No [ ]

16. How old were you when you got married? Age (in years): ✐

17. Have you ever had a pregnancy (including any that did not come to term)?

A. Yes [ ]	B. No [ ]

If **'Yes'** answer Questions 18 and 19. If **'No'** skip to Question 20.

18. How old were you during your first pregnancy? Age (in years): ✐

19. How many children do you have? Write 0 if you don't have any Number: ✐

20. Where do you get your source of drinking water? Select **all** that apply if more than one source?

A. Well (protected)	[ ]
B. Well (unprotected)	[ ]
C. Tap	[ ]
D. Travel to neighbouring village or town	[ ]
E. Other, please specify:	✐

21. When did you last have malaria?

A. I currently have malaria	[ ]
B. Less than 3 months ago	[ ]
C. 3-6 months ago	[ ]
D. 7-12 months ago	[ ]
E. More than a year ago	[ ]
F. I have never had malaria	[ ]

22. When did you last receive deworming tablets?

A. Less than 3 months ago	[ ]
B. 3-6 months ago	[ ]
C. 7-12 months ago	[ ]
D. More than a year ago	[ ]
E. I have never taken deworming tablets	[ ]

23. What is your mother's occupation? Tick **all** that apply if more than one occupation.

A. Housewife	[ ]
B. Small trader	[ ]
C. Gardener	[ ]
D. Secretary	[ ]
E. Teacher	[ ]
F. Doctor	[ ]
G. Nurse	[ ]
H. Lawyer	[ ]
I. Cashier	[ ]
J. Journalist	[ ]
K. Farmer	[ ]
L. Accountant	[ ]
M. Domestic help	[ ]
N. Retired	[ ]
O. Not working	[ ]
P. My mother has died	[ ]
Q. Other, please specify:	✐

24. What is your mother's highest level of education?

A. No formal education	[ ]
B. Primary/ secondary school	[ ]
C. Senior secondary school	[ ]
D. College	[ ]
E. University	[ ]

25. What is your father's occupation? Tick **all** that apply if more than one occupation.

A. Tailor	[ ]
B. Teacher	[ ]
C. Carpenter	[ ]
D. Doctor	[ ]
E. Lawyer	[ ]
F. Small trader	[ ]
G. Merchant	[ ]
H. Gardener	[ ]
I. Nurse	[ ]
J. Farmer	[ ]
K. Journalist	[ ]
L. Driver	[ ]
M. Cashier	[ ]
N. Accountant	[ ]
O. Masoner	[ ]
P. Retired	[ ]
Q. Not working	[ ]
R. My father has died	[ ]
S. Other, please specify:	✐

26. What is your father's highest level of education?

A. No formal education	[ ]
B. Primary/ secondary school	[ ]
C. Senior secondary school	[ ]
D. College	[ ]
E. University	[ ]

Section 3: Community resources

27. What type of food markets do you have in your village or town? Tick **all** that apply

A. Standard build marketplace	[ ]
B. Open market (*Lumo*)	[ ]
C. Supermarket	[ ]
D. Shops	[ ]
E. Street food vendor	[ ]
E. Other, please specify:	✐

28. Do you or your parent/guardian shop for your household food items from your village or town?

A. Yes [ ]	B. No [ ]

If **'Yes'** answer Question 29. If **'No'** skip to Question 30.

29. Why do you or your parent/guardian travel to shop for your household food items? Tick **all** that apply

A. There is no market available in my village or town	[ ]
B. There are limited food choices	[ ]
C. It is cheaper	[ ]
D. Other, please specify:	✐

30. What type of healthcare services do you have in your village or town? Select all that apply? Tick **all** that apply

A. I don't have any in my community	[ ]
B. Hospital	[ ]
C. Health centre	[ ]
D. Clinic	[ ]
E. Pharmacy	[ ]
F. Village health worker	[ ]
G. Traditional midwife	[ ]
H. Other, please specify:	✐

31. What type of roads do you have for vehicles in your community? Tick **all** that apply

A. No pavement [paved] roads for vehicles in my community	[ ]
B. Basalt	[ ]
C. Concrete	[ ]
D. Gravel	[ ]
E. Other, please specify:	✐

32. What type of electricity supply do you have in your village or town? Tick **all** that apply

A. There is no electricity in my village or town	[ ]
B. National Water and Electricity Company (NAWEC) supply	[ ]
C. Private installed solar	[ ]
D. Generator	[ ]
E. Other, please specify:	✐

33. Do you have electricity supply in your household?

A. Yes [ ]	B. No [ ]

If **'Yes'** answer Question 34. If **'No'** skip to Question 35.

34. What type of electricity supply do you have in your household? Tick **all** that apply

A. National Water and Electricity Company (NAWEC) supply		[ ]
B. Private installed solar		[ ]
C. Generator		[ ]
D. Other, please specify:	✐	

35. Do you travel to attend school?

A. Yes, I travel to attend school	[ ]
B. No, I attend school in my village or town	[ ]
C. I am not attending school	[ ]

If **'A',** answer Question 36. If **'B'** or **'C'** skip to **Section 4**

36. Why do you travel to attend school?

A. There is no school available at my village/town		[ ]
B. The school/s cannot accommodate all my peers in the same grade		[ ]
C. Schools outside my village or town are of better quality		[ ]
D. I want to explore other schools in different communities		[ ]
E. My friends are attending that school		[ ]
F. I just like the school		[ ]
G. Other, please specify:	✐	

Section 4: Sources of nutrition information

37. Where do you get information on nutrition-related health issues? Tick **all** that apply.

A. I don't have access to nutrition information		[ ]
B. Family members		[ ]
C. Peers		[ ]
D. School		[ ]
E. Mass media		[ ]
F. Community members		[ ]
G. Health care practitioners		[ ]
H. Other, please specify:	✐	

Section 5: Physical activity

This section is about your physical activity levels in the last seven days (in the last week). This includes sports or dance that make you sweat or make your legs feel tired; games that make you breathe hard, like skipping, running, climbing; watching television, playing games and others. Please remember there are no right and wrong answers (it is not a test). Answer all the questions as honestly and accurately as you can.

38. Physical activity in your spare time: Have you done any of the following activities in the past 7 days (last week)? If yes, how many times? (Tick **one** box per row)

	No	1-2	3-4	5-6	7 times or more
1. Skipping	[ ]	[ ]	[ ]	[ ]	[ ]
2. Walking for exercise	[ ]	[ ]	[ ]	[ ]	[ ]
3. Bicycling	[ ]	[ ]	[ ]	[ ]	[ ]
4. Jogging or running	[ ]	[ ]	[ ]	[ ]	[ ]
5. Swimming	[ ]	[ ]	[ ]	[ ]	[ ]
6. Baseball	[ ]	[ ]	[ ]	[ ]	[ ]
7. Football	[ ]	[ ]	[ ]	[ ]	[ ]
8. Volleyball	[ ]	[ ]	[ ]	[ ]	[ ]
9. Basketball	[ ]	[ ]	[ ]	[ ]	[ ]
10. Dance	[ ]	[ ]	[ ]	[ ]	[ ]
11. Other✐…………………..	[ ]	[ ]	[ ]	[ ]	[ ]
12. Other✐…………………..	[ ]	[ ]	[ ]	[ ]	[ ]

39. In the last 7 days, during your physical education (PE) classes, how often were you very active (playing hard, running, jumping, throwing)? Tick **one** only.

A. I don't do PE	[ ]
B. Hardly ever	[ ]
C. Sometimes	[ ]
D. Quite often	[ ]
E. Always	[ ]

40. In the last 7 days, what did you do at lunch break (besides eating lunch)? Tick **one** only.

A. Sat down (talking, reading, doing schoolwork)	[ ]
B. Stood around or walked around	[ ]
C. Ran or played a little bit	[ ]
D. Ran around and played quite a bit	[ ]
E. Ran and played hard most of the time	[ ]

41. In the last 7 days, on how many days right after school, did you do sport, dance, or play games in which you were very active? Tick **one** only.

A. None	[ ]
B. 1 time last week	[ ]
C. 2 or 3 times last week	[ ]
D. 4 times last week	[ ]
E. 5 times last week	[ ]

42. In the last 7 days, on how many evenings, did you do sport, dance, or play games in which you were very active? Tick **one** only.

A. None	[ ]
B. 1 time last week	[ ]
C. 2 or 3 times last week	[ ]
D. 4 or 5 times last week	[ ]
E. 6 or 7 times last week	[ ]

43. On the last weekend, how many times did you do sport, dance, or play games in which you were very active? Tick **one** only.

A. None	[ ]
B. 1 time	[ ]
C. 2 or 3 times	[ ]
D. 4 or 5 times	[ ]
E. 6 or more times	[ ]

44. Which one of the following describes you best in the last 7 days? Read all five statements before deciding on the **one** answer that describes you.

A. All or most of my free time was spent doing things that involve little physical effort [ ]
B. I sometimes (1 - 2 times last week) did physical things in my free time (e.g. played sports, went running, swimming, bike riding) [ ]
C. I often (3 - 4 times last week) did physical things in my free time [ ]
D. I quite often (5 - 6 times last week) did physical things in my free time [ ]
E. I very often (7 or more times last week) did physical things in my free time [ ]

45. Tick how often you did physical activity (like playing sports, games, doing dance, or any other physical activity) for each day last week.

	None	Little bit	Medium	Often	Very often
Monday	[ ]	[ ]	[ ]	[ ]	[ ]
Tuesday	[ ]	[ ]	[ ]	[ ]	[ ]
Wednesday	[ ]	[ ]	[ ]	[ ]	[ ]
Thursday	[ ]	[ ]	[ ]	[ ]	[ ]
Friday	[ ]	[ ]	[ ]	[ ]	[ ]
Saturday	[ ]	[ ]	[ ]	[ ]	[ ]
Sunday	[ ]	[ ]	[ ]	[ ]	[ ]

46. Were you sick last week, or did anything prevent you from doing your normal physical activities? Tick **one** box

A. Yes [ ]	B. No [ ]

If **'Yes'** answer Question 47. If **'No'** skip to Section 6.

47. What prevented you from doing your normal physical activities? Reason: ✐

Section 6: Sleep and sedentary lifestyle

48. How much time did you spent on your mobile phone during the last seven days (making calls, text, reading etc.)?

A. I don’t have a phone	[ ]
B. Less than 2 hours	[ ]
C. 2 to 4 hours	[ ]
D. 5 to 7 hours	[ ]
E. More than 7 hours	[ ]

49. How often did you watch television (TV) for movies, news, etc. during the last seven days?

A. I don’t watch TV	[ ]
B. I watch TV sometimes	[ ]
C. I hardly watch TV	[ ]
D. I watch TV quite often	[ ]
E. I watch TV always	[ ]

50. How often did you use the internet for social media, reading, or other purposes during the last seven days?

A. I don’t use the internet	[ ]
B. I use the internet sometimes	[ ]
C. I hardly use the internet	[ ]
D. I use the internet quite often	[ ]
E. I use the internet always	[ ]

51. How much time did you spend playing computer or mobile phone games during the last seven days?

A. I don’t have access to computer or mobile phone games	[ ]
B. Less than 2 hours	[ ]
C. 2 to 4 hours	[ ]
D. 5 to 7 hours	[ ]
E. More than 7 hours	[ ]

52. Write down the time you went to bed on each of the school nights last week (answer in hours) and tick AM or PM for each day. Those in English schools provide answers from Sunday to Thursday and those in Arabic schools answer from Tuesday to Friday

	**Time you went to bed**	**AM**	**PM**
Sunday	✐	[ ]	[ ]
Monday	✐	[ ]	[ ]
Tuesday	✐	[ ]	[ ]
Wednesday	✐	[ ]	[ ]
Thursday	✐	[ ]	[ ]
Friday	✐	[ ]	[ ]
Saturday	✐	[ ]	[ ]

53. How many minutes do you think it took you to fall asleep on each of these days (answer in minutes)? Those in English schools provide answers from Sunday to Thursday and those in Arabic schools answer from Tuesday to Friday

	**Time it took you to fall asleep (minutes)**
Sunday	✐
Monday	✐
Tuesday	✐
Wednesday	✐
Thursday	✐
Friday	✐
Saturday	✐

54. Write down the time you woke up on each of these days (answer in hours) and tick AM or PM for each day. Those in English schools provide answers from Sunday to Thursday and those in Arabic schools answer from Tuesday to Friday

	Time you woke up	AM	PM
Sunday	✐	[ ]	[ ]
Monday	✐	[ ]	[ ]
Tuesday	✐	[ ]	[ ]
Wednesday	✐	[ ]	[ ]
Thursday	✐	[ ]	[ ]
Friday	✐	[ ]	[ ]
Saturday	✐	[ ]	[ ]

55. What is the full length of your sleep in each of these nights (answers in hours and minutes example 3 Hours 10 minutes). Those in English schools provide answers from Monday to Friday and those in Arabic schools write from Wednesday to Saturday.

	Full length of your sleep ✐	
	Hours	Minutes
Sunday		
Monday		
Tuesday		
Wednesday		
Thursday		
Friday		
Saturday		

Section 7: Nutrition awareness

56. Of these nutrients numbered 1-4, match them with the foods in the table which are their richest source:

1. Protein 2. Carbohydrate 3. Fat 4. Vitamins

**Food**	**Write the corresponding number/s ✐**
Rice	
Lemon	
Groundnut	
Fish	

57. Which one of the following best describes a balanced diet. Read all four statements before deciding on **one** answer.

A. A balanced diet is a diet that contains fats and carbohydrates only	[ ]
B. A balanced diet refers to eating one type of food group a day	[ ]
C. A balanced diet is a diet that contains the ten main food groups required for body building	[ ]
D. A balanced diet is a diet that is made of the required amount of nutrients needed in a day and this include the six main nutrients: fat, protein, carbohydrates, fibre, vitamins, and minerals	[ ]

58. Which one of the following do you associate with overweight. Read all four statements before deciding on **one** answer.

A. Eating carbohydrate foods (e.g. garri, rice) at night can lead to overweight	[ ]
B. Eating diverse food groups can lead to overweight	[ ]
C. All the above can lead to overweight	[ ]
D. None of the above can cause overweight	[ ]

59. Do you think eating sour food like citrus fruits first thing in the morning can cause underweight?

A. Yes [ ]	B. No [ ]

Section 8: Nutrition related cultural rules

60. Which of the following rules do you have in your community? Tick **all** that apply.

A. Adolescents should not take items from the middle of the bowl when eating with elders, they should wait to be given by the oldest person they are eating with	[ ]
B. Adolescent school goers should not eat the head of a fish	[ ]
C. Pregnant adolescents should not eat certain foods such as banana, sea snails or bitter tomatoes	[ ]
D. When serving the family, the greater portion of the good fish/meat/chicken should be given to the men	[ ]

61. Do you think any of the rules identified above can affect your nutritional intake in terms of **quantity**?

A. Yes [ ]	B. No [ ]

If yes, list the corresponding letters (A,B,C,D) from Question 60 for the rules that you think affect **quantity**:	✎

62. Do you think the rules identified above can affect your nutritional intake in terms of **quality**?

A. Yes [ ]	B. No [ ]

If yes, list the corresponding letters (A,B,C,D) from Question 60 for the rules that you think affect **quality**:	✎

THANK YOU FOR COMPLETING THE QUESTIONNAIRE

Factors influencing malnutrition among adolescent girls in The Gambia: A mixed-methods pilot study. *Text in red denote modifications to the questionnaire based on study findings.
