# Supplementary material for: Factors influencing malnutrition among adolescent girls in The Gambia: a mixed-methods exploratory study
Source: BMC Public Health. 2025 Jan 8;25:80. doi: 10.1186/s12889-024-21242-w (PMC11708179; doi:10.1186/s12889-024-21242-w)
Supplement: Supplementary file 4 — Supplementary Material 4. A. Additional quotes supporting the qualitative analysis and B. Focus group memos [file 12889_2024_21242_MOESM4_ESM.docx]

**A. Additional quotes supporting the qualitative analysis**

| **Main theme/ subtheme** | **Additional quotes** |
| --- | --- |
| **1. Cultural norms: harmful vs beneficial to nutrition related health**  Harmful practices  Beneficial practices | *‘When we are eating, if our hands touch the middle of the bowl, our Aunt usually beat them; You know my younger sibling like chicken very much, so when my aunt prepare chicken, she will bury the chicken in the middle of the bowl covered with rice and put hot pepper on top of it so that we will eat the hot pepper when we touch the middle of the bowl’ (P24, urban, 15 years).*  *‘Concentrating where they are eating is good because it will help them eat well and they will know what ingredients to take’ (P32, urban, 11 years).* |
| **2. Concepts of healthy diet and weight**  Certain food groups and nutrients are healthy  Overeating leads to obesity  Underweight and overweight caused by illness | *‘Healthy diet comprises eating rice with little bit of salt added to it with vegetables such as cucumber, carrots and tomatoes’ (P31, urban, 10 years).*  *‘Eating combination of egg, rice, beans is nutritional health’ (P3, rural, 14 years).*  *‘Overweight and obesity is caused by eating healthy foods’ (P26, urban, 16 years)*  *‘Not eating healthy foods’ (P27, urban, 17 years).*  *‘Eating rice and protein causes overweight and obesity’ (P28, urban, 15 years).*  *‘If you eat uncovered contaminated food that can cause sickness which can cause undernutrition’ (P24, urban, 15 years)*  *‘Some people are overweight/obese because they are sick, the weight is caused by diseases’ (P21, urban, 16 years).* |
| **3. Approaches to tackling under- and overnutrition**  Individual responsibility  Household responsibility  Collective action in communities  National solutions | *‘The individual should eat healthy foods like fruits and vegetables to help build up the body’ (P23, urban, 17 years).*  *‘If someone is overweight/obese and want to reduce it, that person should drink lime first thing in the morning before eating and in the evening before going to bed but should not drink it during the day (midday) and that individual should also be preparing and eating sour foods’ (P10, rural, 14 years)*.  *‘They should drink hunger medication supplement – appetite boro, domor boro [commercial appetite stimulants] – that increase appetite’ (P12, rural, 16 years).*  *‘If someone is overweight/obese and wants to reduce their weight, they should drink something like medications to reduce their weight. My friend was obese and she started drinking a certain type of tea, like slimming tea every day and doing exercise after drinking it. She has now reduced weight’ (P24, urban, 15 years).*  *‘Parents should have access to loans when they need money. For example some parents are farmers and their source of income is seasonal, so is important to get access to money when their children are sick or they want to make small scale business and return that money later when their harvest are ready’ (P21, urban, 16 years).*  *‘If parents are aware of good nutrition then they will tell their adolescents to eat good food and prevent foods that cause overnutrition’ (P15, rural, 17 years).*  *‘Rich households will throw their excess away either food or not…people should be kind to each other and should be sharing when they are better off’ (P24, urban, 15 years).*  *‘Some parents have hypertension or fatty foods in their body, and they are usually advised by Doctors and Nurses to exercise, they can advise their children to exercise with them’ (P23, urban, 17 years).*  *Parents should be very observant about what their children eat, but some parents don’t pay attention to this at all. Parents should also ensure adolescent get medical attention when they have nutrition issues’ (P32, urban, 11 years).*  *‘Community leaders provide food for adolescents’(P1, rural, 13 years).*  *‘Community members tell adolescents to reduce the amount of food eaten’* *(P8, rural, 14 years).*  *‘Community leaders should encourage nutrition sessions among adolescents in schools and in the community through collaboration with nutrition and health experts’ (P32, urban, 11 years).*  *‘They should provide free housing for those who don’t have houses’ (P24, urban, 15 years).*  *‘Adolescents in the community should be provided with free access to playgrounds, swimming pools, training fields for them to use. We don’t have free access to playgrounds and swimming pools in our estate, those available are found in private schools where only students in those schools have access’ (P24, urban, 15 years).*  *‘The community should provide empty land to adolescents where they can do gardening, rear animals, and do other activities’ (P21, urban, 16 years).*  *‘The government should help us fence our gardens and access to water, fertilisers and seeds so that we can grow enough to eat and sell’ (P9, rural, 17 years).*  *‘Provide adolescents with medicines and advise their parents to give them good food’ (P29, urban, 16 years).*  *‘Adolescents provided with adequate tools and resources for farming and rearing animals like sheep or poultry; They should also be taught to adopt the culture of rearing animals in their homes’ (P19, urban, 11 years).*  *‘Government educate adolescent and ensure access to information in homes or in schools about good or bad foods’ (P15, rural, 17 years).*  *‘The government should encourage schools to conduct PE at least once a week. Right now, PE is stopped in our school because of Covid-19 and we are not having it at all’ (P19, urban, 11 years).*  *‘The government should help adolescents with medications that can reduce their weight’ (P32, urban,11 years).* |
| **4. Recruitment barriers and facilitators**  Parental influence *vs* autonomy  Diverse practical strategies needed  Building trust  Participation not guaranteed | *‘Explain purpose of research to parents and this will make them agree’ (P8, rural, 14 years)*.  *‘After discussion and agreement with the parents, participants will come by themselves to the meeting point when they see the researchers in the community’ (P5, rural, 10 years).*  *‘Some children may face with problem of dilemma but take your time to explain to them so that they can understand better’*. *(P23, urban, years).*  *‘You can also go to the schools in this community and ask for people who reside within this setting and share the information with them. These people will also share the information in their surroundings’ (P19, urban, 11 years).*  *‘You know it depends, some people don’t trust strangers, some people may not be interested even to listen to you in the first place; Some people also believed in privacy and they think that they should be contacted first for example by calling before coming to their houses in person’ (P19, urban, 11 years).*  *‘Some adolescents will not participate even if they are told by their parents to participate and if they are compelled by their parents to participate, they may not be engaged in the discussion’ (P32, urban, 11 years).* |
| **5. Survey questionnaire and proposed measures are mostly feasible and acceptable**  Follow up strategies: remote methods may be challenging | *‘Some of our parents do not allow us to use mobile when going to school especially the younger ones less than 15 years’ (P17, urban, 11 years).*  *‘The network quality depends on the area the participant is’ (P25, urban, 12 years).* |

**B. Focus group memos**

**Focus Group Discussion (FGD1)- Rural area I**

- **Cultural norms and practices**
- **What are the cultural norms and practices?**

A child should not touch the fish or meat in the middle of the bowl when eating with elders-P3

A child should not get used to picking food from the middle of the bowl because they will behave similar in other houses or ceremonies and that can cause attention to the child by other people which can lead to shame to the parent-P1

When dishing food, men will get the first and best share of the food like the biggest fish, most of the meat, followed by adult women and the children will get the last and least share of the fish or meat- P1-P8

Eating too much is associated with stigmatisation and greediness and those people are termed as ‘Habu’. Any place they go, they will be the topic of discussion and this culture has forced many people in the community to eat less- P1-P8

School-goers should not eat the head of a fish because it will make them dull in school-P1-P8

Children eating between lunch and dinner ‘Sita’ is encouraged in our community-P1-P8

- **How does these cultural norms and practices influence nutritional level of adolescents?**

If you tell adolescents not to eat meat or fish from the middle of the bowl, it can make them have less weight and can even make them sick- P4

Not eating enough meat or fish can make you lose weight-P8

If you prevent adolescents from touching the middle of the bowl, they can lose weight- P1, P2 and P3

- **Which of the cultural norms and practices are beneficial?**

Eating between meals will make you healthy and have good weight-P1

- **Which of the cultural norms and practices are harmful?**

Preventing children from touching the middle of the bowl can be harmful to them and reduce their weight-P4

- **Views on diet, underweight and overweight**

Eating egg is good health-P2

Eating rice is good food-P1

Eating combination of egg, rice, beans is nutritional health-P3

Eating meat is nutritional health-P4

Eating banana and apples is good for health-*P5

Eating chicken, cous cous, egg is nutritional health-P6

Eating potatoes, oranges is nutritious-P7

Eating beans, omellette, mashed potato-P8

- **Reasons for underweight**

Not eating enough food-P1-P8

- **Prevention of underweight**

If you eat good food, you will not be underweight-P7

If you eat you will not be underweight- P3

You should eat clean food and enough food to prevent your weight from going down-P5

You should eat well, drink water, wash hands before eating and relax well to get good weight-P6

You should eat at a clean environment and avoid illnesses like diarrhoea and stomachache-P7

You should eat in a clean environment and wash hands before eating-P2

If you eat well, you will not be underweight-P4

You eat good food and enough food and cover food items well to prevent flies from entering the food.

- **Reasons for overweight/obesity**

Eating too much causes overweight/obesity-P3

Eating too much and eating constantly is the reason for being overweight/obese-P6

A person who spent too much time eating and does not feel satisfy becomes overweight and obese-P8

Eating different types of foods like bread, rice and porridge at short intervals can cause overweight/obesity but if you eat one type of meal that can reduce the risk of being overweight/obese-P8

Eating food like bread whilst lying down causes overweight/obesity-P5

If the individual sit at the bowl to eat, they spent long time eating because they don’t feel satisy-P7

- **Prevention of overweight/obesity**

Reduce the amount of food eaten-P3

Reduce the amount of food eaten and exercise to reduce weight-P4

The person should exercise, eat good food like banana and stop eating too much rice, dinner and eating rice for dinner-P1

The individual should reduce eating at night -P5

They should reduce eating dinner-P6

They should reduce the amount of food eaten-P7

They should wait for a while after eating dinner before going to bed-P8

- **Composition of a healthy diet**

Healthy diet consists of coos ‘Njeleng’, meat with ‘domoda’-P3

Healthy diet composed of ‘mono’ (porridge) with sugar and sour milk-P4

Healthy diet is ‘Futo and Dajiwo’ (Ingredients consist of pumpkin, beans, fish, peanut)-P7

Benachin with meat, beans and ‘kucha’, is healthy diet-P6

Palm oil stew with fish ‘furo’, potatoes, bitter tomatoes, cabbage, ‘Kucha’ is healthy diet-P2

Caldou with fish, mutarde, onions, black pepper, jumbo, garlic by boiling but add small oil is healthy diet-P1

Domoda is a healthy diet if it has meat, cabbage, bitter tomato and pepper-P5

‘Superkanja’ (Okra soup is a healthy diet with bitter tomato, okra, smoked catfish, bigger pepper, palm oil).

- **Perceived weight status**

Normal-P3 Underweight-P5

Normal-P1 Underweight-P6

Normal-P2 Normal-P7

Normal- P4 Normal-P8

- **Views on addressing female adolescent malnutrition**
- **Undernutrition- National level**

The government should provide communities with food every month-P8

The government should give food to our communities-P7

- **Undernutrition-Community level**

Community leaders should provide rice and cooking utensils for households-P1

- **Undernutrition- Household level**

Adolescents should be engaged in family food preparation and cooking in the household-P6

Adolescents should be provided with money when going to school so that they are able to buy food at school-P6

Family heads should provide fish money for the family-6

They should be provided good food-P7

- **Overnutrition- National level**

The government should reduce food assistance- P5

The government should provide food assistance-P2

- **Overnutrition-Community level**

Community leaders Provide food for adolescents-P1

Community members should tell adolescents to reduce the amount of food eaten- P8

Community members should help adolescent reduce food intake ‘if they eat in the morning, they should skip the next meal’-P5

- **Overnutrition- Household level**

Mums should help adolescent reduce weight but don’t know how-P7

They should reduce eating-P6

Parents should help children reduce eating too much food-P6

If parents agree to it then the participants will also agree-P1,2,3 and P4

- **Engaging adolescents in research**

- **Method:**

Make a call before coming-P1

Contact and inform the parents and guardians of the participants- P1-P8

Make a meeting at the community centre with parents and guardians to discuss about the research- P1-P8

Meet parents in their respective homes-P1and P8

Take time to talk to parents to convince them for their children to participate-P4

Explain purpose of research to parents and this will make them agree-P8

- **How to implement above method:**
  Make a meeting with all parents at the Bantaba- P1-P8

Search house to house for parents who do not attend the meeting at the Bantaba- P3

- **Other methods**

Contact individual parents and discuss- about the research -P6

- **Access to participants in community:**

After discussion and agreement with the parents, participants will come by themselves to the meeting point when they see the researchers in the community -P5

If participants did not come at the meeting point, then the researcher should go to their individual houses to look for them-P5

- **Challenges to recruit participants:**

There can be delay in getting participants on time at the meeting point-P2

Some parents may refuse for their children to take part in the study even after discussing with them-P7

Some participants may be engaged in domestic work which can prevent them from participating in the research-P1

- **Motivation factors-Parents:**

Talk to parents and use convincing techniques for parents to agree-P4

Some parents may refused because most girls will be engaged in domestic work at home, so you need to take your time, explain the importance and benefits of the research to be able to convince them-P1

If you explain the procedure of the research and what it entails to the parents, they will agree-P8

- **Motivation factors-Participants**

If parents agreed to the research, then all participants will agree-P1-P8

If parents agreed to the research, then all participants will agree because it is against our culture to go against your parents’ words-P7

We all don’t have mobile-P1-P8

You can contact our peers through their parents’ mobiles or the Village Development Committee (VDC) focal person-P1-P8

- **Best network**

Africell- P8, 7, 4 and P3

Comium-P6 and P2

Q-cell- -P5 and P1

- **Can network quality accommodate conversation**

Yes-P1 -P8

- **Alternative method to meet participants**

Researcher should come back and meet participants in person- P1-P8

- **Willingness to provide blood and urine sample**

Participants will accept when they see their peers doing it-P8

Participants will accept-P7

Participants will accept if the researcher informed their parents prior and gained their consent-P5 and P6

- **Completing the questionnaire**

Participants said the questions in the questionnaire were generally not difficult but the sedentary lifestyle section where they indicated the time they went to bed and the time they wake up was challenging because they don’t normally have access to a device to check time and most of it was an estimation-P1-P8.

NB: All the questions were interpreted to all the participants.

**Focus Group Discussion (FGD2)- Rural area II**

- **Cultural norms and practices**
- **What are the cultural norms and practices?**

Adolescents should not eat lime and pregnant adolescent should not eat eggs or drink cold water. The egg will make the baby dumb-P9-P16

Adolescents are advised to eat egg, ‘Futo’#, Findi and rice-P10-P16

Usually, adolescent girls are told in our society not to eat ‘Pura’ (Penguins) because it can prevent them from getting breastmilk during breastfeeding-P10

We are told a pregnant lady should not eat ‘Kacho’ (a type of bird) because the baby will grow to become a talkative-P12

Adolescents should not eat pepper because it can cause ulcer and pregnant adolescents should not watch movies with certain creatures in it because the baby can have similar features when born-P13

If you are not married, you should not have sexual relations with boys because you can bring problems to your parents that you cannot be free of-P15

When an adolescent girl is eating with elders, she should be putting the ingredients at their side as they eat because it is a sign of respect for that person-P14

We are told school-goers should not eat the head of the fish because it will make them dull in learning-P9-P16

When dishing, the best part of the food goes to the men’s bowl.

- **How does these cultural norms and practices influence nutritional level of adolescents?**

The best part of food going to Men’s bowl will not affect girl’s share of the food-P10-P16

- **Which of the cultural norms and practices are beneficial?**

If an adolescent with high haemoglobin level is told not to eat egg that is a good practice because it will lower their blood level by not eating it-P15

- **Which of the cultural norms and practices are harmful?**
- **Views on diet, overweight and underweight**

Eating meat is nutritional health-P9

Nutritional health means cleanliness in cooking and its surroundings and the food should have fish or ‘Nada’ dish which includes (Jumbo, pepper, tomato, fish, bitter tomato, vegetables, ‘Mafu Jaroh, Netatu)-P10

Nutritional health means eating rice and ‘Teya dourago’ which has tomato, salt, pepper, shrimps and bitter tomato-P11

Eating ‘Njangkatago’ with groundnut, Jumbo, ‘Mafo’, Netatu, Smoke fish and rice is nutritional health-P12

Eating ‘Kucha’ with smoke fish, Jumbo, salt, Bitter tomato, ‘mafo’ and sorrel by boiling without adding oil is good nutrition-P13.

Eating ‘Superkanja’ (okra soup) that consist of pepper, ‘mafo’, smoked fish, jumbo and smoked cat ish is good nutrition-P14.

‘Superkanja’ is good for health and it usually has ‘Ndambu’ (‘kerengkereng’), okra, bitter tomato, Jumbo, fresh and dried fish, smoked fisk, smoked cat fish, pepper and ‘netatuwo’-P15.

Eating white ‘Benachino’ is good for health and it usually has meat, bitter tomato, cabbage, ‘Adja koyoh’, salt, pepper and Jumbo-P16

- **Reasons for underweight**

If the individual did not eat good food their weight will drop down-P16

If the person did not eat good food like apple, egg, banana and corn they will be underweight-P15

- **Prevention of underweight**

The person should eat potatoes, ‘Njekoyo’ (white fish) and mango that is good for body-P14

Adolescents should eat ground coconut, mango, egg, banana, ‘Dougoula’ and ‘Mono’ to prevent underweight-P13

They should drink medications (‘appetite boro’; ‘domor boro’) that increase appetite-P12

Underweight person should eat ‘Garri’, cornflakes and make ‘ponsehwo’ at night (bread mix with sugar and water and on some occasions milk added) to increase weight-P11

If the individual eat good food and do exercise they can have good weight-P10

- **Reasons for overweight/obesity**

I don’t know the causes-P9

Some diseases like Hypertension can cause overweight and obesity-P10

If someone does not eat good food e.g. if you found something like a Mango in a place and eat it that can cause overweight and obesity-P9

If someone is overweight/obese that is natural sometimes and can also be God making-P8

If someone eat mango and throw it and another person pick that same mango and eat it like that then that can cause overweight and obesity-P13

If you found open food with flies and eat it like that that can cause overweight and obesityP14

Eating good food can cause overweight and obesity but overweight and obesity is good health-P14

People can be born with overweight and obesity and sometimes is God making. Some people also eat too much and almost everything they see and this can make other people put poison in their food which can cause their overweight and obesity-P15

Sometimes you see babies born very thin but when they are given powder potato (‘pompeter munko’) their weight increases-P16

- **Prevention of overweight/obesity**

If you are big and you want to reduce your weight, you can do exercise-P9

If someone is overweight/obese and want to reduce it, that person should drink lime first thing in the morning before eating and in the evening before going to bed but should not drink it during the day (midday) and that individual should also be preparing and eating sour foods-P10

Someone can prevent overweight and obesity by running-P11

Doing domestic work and gardening can prevent overweight/obesity-P12

Sickness can also prevent overweight/obesity because it can reduce too much eating-P13

Doing exercise can prevent overweight/obesity-P14

Engaging in trainings and exercises like running in the field and lying on the ground doing heavy iron lifting can prevent overweight and obesity-P15

- **Composition of a healthy diet**

Healthy diet comprises cucumba mix with mayonnaise and eat it with bread-P10

Healthy diet is mashed potato mixed with mayonnaise, add egg and jumbo)-P9

Eggs gives good health-P14

Boiling sweet potato and peeled the skin before eating it is a good diet-P12

Eating apple and coconut is healthy diet-P13

If you buy sardine from the shop and mixed it with corned beef, eggs,salad,vinegar carrots, cucumba and mayonnaise and a cup of tea with sugar and milk is a healthy diet-P14

Eating grilled meat with onions, cucumba and mutarde is healthy diet-P16

- **Perceived weight status**

Normal-P9-P16

- **Views on addressing female adolescent malnutrition**
- **Undernutrition- National level**

The government should help the adolescents with good food like rice and cooking oil-P10

They should be provided with rice and cooking oil-P11

The government should bring rice, cooking oil, onions, potatoes and eggs-P12

The government should help us fence our gardens and access to water, fertilisers and seeds so that we can grow enough to eat and also sell-P9-P16

They should help communities like ours with electricity, good road, standard market and health facility. We walk for long distance to go to MRC Keneba-P9-P16

- **Undernutrition-Community level**

The community should provide good food for adolescents-P14

Community leaders should make sure whatever assistance that come from the government like foodstuffs reached the adolescent girls-P9-P16

- **Undernutrition- Household level**

Adolescents should appease their parents so that they are able to get money from them to buy good food to eat-P10

Parents give adolescents foods that have vitamins-P16

Parents should provide adolescents with good food to eat-P15

- **Overnutrition- National level**

The government should provide adolescent with good food-P14

Government should educate adolescent and ensure access to information in homes or in schools about good or bad foods-P15

- **Overnutrition-Community level**

If there is a health facility in the community, adolescent girls can go there to check their weight and get advised on good nutrition-P9

- **Overnutrition- Household level**

Parents should advised their children not to eat rice at night-P16

If parents are aware of good nutrition then they will tell their adolescents to eat good food and prevent foods that cause overnutrition-P15

- **Engaging adolescents in research**
- **Method:**

If the participants have a good experience from the pilot study and learnt something relevant from it then parents will allow their children in subsequent ones-P10

Our colleagues will be willing to participate in future studies-P9-P16

After this interview, we will inform the rest of our peers in the community and the benefits in participating and this can encourage them to participate in the main study-P15

The participants agreed that to get high turnout, the research should be conducted at the ‘Bantaba’ (village meeting point) and the participants will come there by themselves or the VDC focal person will mobilise them-P9-P16

Explain the importance of the research to parents- P10

- **How to implement above method:**
- **Other methods**
- **Access to participants in community:**

Their experience with the pilot study whether good or bad will determine future recruitment-P9-P14

- **Challenges to recruit participants:**

Participants will take part in the main study if it is relevant to them-P15

If the research involves blood and urine collection, people will agree to participate but if it involves injection they will not take part because of issues surrounding Coid-19-P12 and P13

- **Motivation factors-Parents:**

You will tell parents the work is not to harm children and they will benefit in participating-P15

- **Motivation factors-Participants**

If our current experience is good and the research is important then the rest will also follow suit-P13, P15 and P16

- **Willingness to provide blood and urine sample**

Since the research is about health, that alone can motivate participants to succumb to blood and urine sample collection-P15

If the participants for the pilot study has good experience then that will motivate others to participate-P14

If the sample collection is associated with health then they will agree-P13

- **Participants access to mobile phones**

We all don’t have mobile phones and our peers don’t have too; we used our mother phones for communications sometimes -P9-P16

You should buy mobile for the participants so that you can get them directly-P10

If you call some of them their mother’s, you can get them-P9

You should buy mobile for participants to ease communication-P11

You can call those who have phone directly on their mobile-P12

Call mothers to get access to adolescents-P13 and P14

Buy mobile phones for them-P15 and P16

- **Best network**

Q-cell-P9

Africell- P12, P13, P14 and P15

Qcell and Africell- P10, P11 and P16

- **Can network quality accommodate conversation**

It depends, sometimes the network is good and sometimes is bad-P9-P16

- **Alternative method to meet participants**

Researcher should come back to the community if they cannot access the participant. They can also find a representative who can conduct the interview with the participants-P14

They should take the number of their parents and call the participant to communicate-P11

The researcher can call the VDC focal person who is well known in the community to talk to the participants-P12

They can also call mothers mobile phones to access participants-P13

They can call the participant through their fathers’ phone-P15

- **Completing the questionnaire**

The questions were not generally bad.

NB: All the questions were interpreted to most of them because majority were attending Arabic schools and did not understand some of the questions very well.

**Focus Group Discussion (FGD3)- Urban area I**

- **Cultural norms and practices**
- **What are the cultural norms and practices?**

Pounded white rice mixed with water and sugar should not be eaten by adolescent girls because it will make their hymen white during their first sexual intercourse with their future husband-P17

Pregnant adolescent girl should not eat ‘Kew’ and bitter tomatoes because it will affect the baby when born. They said when a baby is born with rashes that is usually caused by the mother eating bitter tomatoes when pregnant-P23

As an adolescent girl, it is advised not to get used to bending your toes because that is a signpost of burying any man you get married to in the future-P17

When an adolescent is eating with elders, their hands should not reach the middle of the bowl-P19

When an adolescent is eating with elders, the left hand should hold the bowl and should not talk-P22

An adolescent should be focused on what they are eating, if not something can enter in the food without noticing and they eat that it can cause harm-P19

When we are eating, if our hands touch the middle of the bowl, our aunt usually beat them. You know my younger sibling like chicken very much, so when my aunt prepare chicken, she will bury the chicken in the middle of the bowl with rice and put hot pepper on top of it so that we will eat the hot pepper when we touch the middle of the bowl-P24

Children should hold the bowl when eating with elders-P18

- **How does these cultural norms and practices influence nutritional level of adolescents?**

If an adolescent is asked not to touch the vital ingredients placed in the middle of the bowl can cause stunted growth because those ingredients and proteins are important for their growth-P19

If someone concentrate on the food whilst eating can help in growth because it can make the individual eat the adequate amount of food-P23

Putting pepper in the middle of the bowl is not good because it can expose the person to ulcer if practice frequently-P19

Asking adolescent not to eat pounded rice with sugar is bad practice because the rice and sugar have carbohydrate and energy which is important to them-P17 and P19

Adolescents should not be told not to eat bitter tomatoes because it has vitamins necessary for their growth-P21

- **Which of the cultural norms and practices are beneficial?**

Focusing on the food when eating-P23

- **Which of the cultural norms and practices are harmful?**

Not to eat pounded white rice- P17 and P19

Not touching the middle of the bowl-P19

Not to eat bitter tomatoes-P21

- **Views on diet, underweight and overweight**

It refers to good food that can cure certain ailments in your body-P23

It helps in body building and can lead to healthy body-P18

Is nutrition that is good for the body and helps you get healthy body-P19

It can be food that contain vitamins and proteins-P17

Nutritional health is when you eat good food for your body and it makes your body healthy-P20

It refers to nutrition that causes illness or wellness for example some people when they eat ‘Benachin’ and their body does not accept it, they can become sick but some people can grow from it-P24

Nutritional health can be good or bad. Some foods can be good and some foods may be bad-P22

- **Reasons for underweight**

Lack of some vitamins in your body can cause underweight-P23

If you are not getting the required amount of food in your body-P21

Underweight can be cause if someone is eating the food they should not eat and they are not eating the food they should eat like body building foods-P17

If you eat uncovered contaminated food that can cause sickness which can cause undernutrition-P24

- **Prevention of underweight**

If a person is not used to going to bed after eating, then that person will not gain weight. If someone wants to gain weight, they should go to bed immediately after eating-P24

The individual should eat healthy foods like fruits and vegetables to help build up the body-P23

They should eat healthy foods like vegetables and fruits and avoid dirty environment-P19

You know if you eat and go to bed and eat food that increase weight like rice and chicken stew you can have weight-P20

Eating ‘Superkanja’ (okra soup) can increase weight-P22

There are certain drugs when you buy them and drink it you can have weight like ‘super apeti’. They can be either tablets or syrup-P18

Some people also said eating at night can increase weight-P21

Eating foods that does not contain oil like ‘Bisap’ can helps gain weight-P20

- **Reasons for overweight/obesity**

If you eat and did not wait for the food to digest and you lie down that can cause overnutrition-P19

If you are eating too much and you are not exercising your body like doing household chores can cause overweight and obesity-P22

Overnutrition can be because of accumulation of fats like eating Ice cream-P23

Some people are overweight/obese because they are sick. The weight is caused by diseases-P21

- **Prevention of overweight/obesity**

The person should be doing exercise-P23

The individual should be doing exercise, dieting and reduce fatty foods like oily foods-P19

Going to the field and doing exercise can reduce weight-P20

If someone is overweight/obese and wants to reduce their weight, they should drink something like medications to reduce their weight. My friend was obese and she started drinking a certain type of tea like slimming tea everyday and doing exercise after drinking it. She has now reduced weight-P24

You can go for exercise to reduce weight-P22

You can exercise to reduce weight-P18

- **Composition of a healthy diet**

Is a diet that contain fruits and vegetables-P23

Foods that contains vitamins and proteins-P19

Eating whatever you want-P20

If the food does not contain cooking oil or palm oil is a healthy diet-P24

Eating food like ‘Bisap’ that have Jumbo and ‘netatu’ is healthy diet-P22

You can classify it as foods that contain vitamins and proteins like beans-P17

Some people said if you want to eat healthy foods you should eat cucumba, salad, vegetables and carrots-P21

Eating plenty Jumbo is not good but you can replace it with salt and pepper-P18

- **Perceived weight status**

Normal-P18 Normal-P21 Underweight-P17 Normal- P22 Underweight-P24

Underweight-P20 Normal-P19 Overweight-P23

- **Views on addressing female adolescent malnutrition**
- **Undernutrition- National level**

The government should educate both parents and adolescents about good nutrition-P23

The government should put in strategies to educate parents in the communities about good nutrition because some parents are not educated and are unaware of good nutrition. In return, they will be able to help their adolescents eat good nutrition-P19

Older adolescents should be provided with job opportunities so that they will be able to afford good food-P20

The government should help adolescents with free school meals because the food sold at school is expensive and sometimes when you buy it you can find a foreign body in it which means you wasted money because you will not eat it-P24

Some food sellers bring leftover foods the following day if that food was not completely sold out the previous day and this can be poisonous to children-P19 and P24

Government should give money to parents at home so that they are able to prepare adequate dishes for children-P22

The government should help adolescent with food or money through the district chief or Alkalo who will distribute that aid to the community members-P17

You know some adolescent when they complete school they don’t have jobs, the government should help them get jobs-P21

The government should help adolescents eat good food everyday by providing their parents with money to buy foodstuffs-P18

Adolescents should be provided with resources to enable them do vegetable gardening in their backyards or at school-P23

The adolescents should be provided with the adequate tools and resources for farming and rearing animals like sheep or poultry. They should also be taught to adopt the culture of rearing animals in their homes-P19

There should be free access to foods for adolescents which the government should pay for. For example, adolescents can take foods from supermarkets and the government pays for it-P24

Schools should be provided with money to prepare good food for students-P22

- **Undernutrition-Community level**

Community leaders should provide adequate information on good nutrition and the different nutrients necessary for growth for adolescents-P23

The community should provide empty land to adolescents where they can do gardening, rear animals, and do other activities-P21

They should provide free housing for those who don’t have houses-P24

- **Undernutrition- Household level**

Parents should be engaged in paid jobs that will earn them money to be able to cater for their children-P22

If a parent is working as a maid in a compound and they have excess food, they should share that food with the maid to take for their children. I watch a movie yesterday about two families; a rich family and a poor family but the rich family does not share anything with the poor family. They will throw their excess away either food or not. Later things changed, the rich family became poor and lived in a poor house whilst the poor family became rich and lived in a beautiful house. What I learnt about the story is that people should be kind to each other and should be sharing when they are better off -P24

The parents should provide good food for the children and make sure they ate enough-P20

Parents should have access to loans when they need money. For example some parents are farmers and their source of income is seasonal, so is important to get access to money when their children are sick or they want to make small scale business and return that money later when their harvest are ready-P21

- **Overnutrition- National level**

The government should educate adolescents about the causes of overnutrition-P23

Government should provide adolescents with access to free gym without paying-P22

The government should encourage schools to conduct PE at least once a week. Right now PE is stopped in our school because of Covid-19 and we are not having it at all-P19

Even field trips are stopped because of Coronavirus, the government should help us reduce the Covid-19-P24

- **Overnutrition-Community level**

Adolescents in the community should be provided with free access to playgrounds, swimming pools, training fields for them to use-P24

We don’t have free access playgrounds and swimming pools in our estate, those available are found in private schools where only students in those schools have access-P24

- **Overnutrition- Household level**

Some parents have hypertension or fatty foods in their body, and they are usually advised by Doctors and Nurses to exercise, they can advised their children to exercise with them-P23

Parents should restrict adolescent from eating junk food-P19

The adolescent can go along with their father to the field to exercise-P20

Parents should encourage their adolescents to walk when going to school-P18

Some adolescents when going to school even a short distance they will take a car. They should be encouraged by their parents to walk to school if the school is not far-P22

Parents should let their children to be exercising at the beach-P21

- **Engaging adolescents in research**
- **Method:**

You will come and share the information with someone you know in the community and that person will spread the details in the community-P17

You will visit the compounds one by one and explain the reasons for the research to individual households and these people will also likely share the information in their neighbourhoods-P23

You can also go to the schools in this community and ask for people who reside within this setting and share the information with them. These people will also share the information in their surroundings-P19

You can go in the community for example the Alkalo and inform them about the research and they can conduct meetings with the parents and this will give you access to adolescents in the community-P17

- **How to implement above method:**

For example, you know you are here, and you already know someone in the community that person can direct you to members in the community-P17

When you visit the houses, you explain the purpose of the research and if parents are interested, they will let their children participate but sometimes the child may reject after parent consenting so is important to discuss with them too-P23

In the schools, you can talk to the principal to gain access. You know students usually have extracurricular activities during school hours, the principal can give you that period to speak to the students directly and some of them will be willing to join and spread the information to their peers in the community-P19

- **Other methods**
- **Access to participants in community:**

If you come to the community, you can talk to anyone you meet on the street and ask them about the location you want to go for example school after explaining the research. Some people may not respond when you approach them but some will-P23

You should go to the compounds and talk to them-P21

When you visit the houses, you should ask for the mums in the house and talk to them so that they can allow their children to participate-P20

You can also visit crowded places in the community like mosques where you can meet the Imam and discuss about the research and the people in the mosque can also share the information further in the community-P19

If you come and talk to parents about the study, they will ask for your identity and some people you know in the community and if they trust you (the researcher), they can allow their children to participate-P22

- **Challenges to recruit participants:**

When visiting compounds, is important to go with someone who knows the household members to gain easy access. If you don’t know someone who knows the household members you can go alone and if they reject you can go from one house to another-P24

Lot of misunderstanding and delays may happen before some people will accept to participate in the research especially if they don’t recognise you as member of the community. Some may even close their doors behind you when you approach them. Is important you talk to the Alkalo to gain access-P17

You know it depends, some people don’t trust strangers, some people may not be interested even to listen to you in the first place. Some people also believed in privacy and they think that they should be contacted first for example by calling before coming to their houses in person-P19

If you come some may accept but others may also reject to participate-P22

- **Motivation factors-Parents:**

As I have explained earlier, when you meet parents tell them about the project in details and that can make them allow their children to participate-P23

You know some parents usually advised their children not to eat certain foods but their children do not follow their advised, so when someone outside come to discuss nutritional issues to their children they will be willing to let them participate because they are aware and knows the importance of nutrition and health-P19

Discuss the purpose of the research to mums-P20

The parents will ask whether the study is beneficial, If they know that it is beneficial for their children then they will let them participate-P24

- **Motivation factors-Participants**

You go and discuss with the parents together with the children and if they agree they will participate. You engage children in the discussion process-P22

When you go you talk to the children politely and explain to them the benefits in participating-P24

Some children may face with problem of dilemma but take your time to explain to them so that they can understand better-P23

You will explain to them is about their health and that can encourage them to participate-P19

- **Willingness to provide blood and urine sample**

Some will be willing for blood and urine samples collection because they have health issues-P23

Not all will be willing to get their blood or urine collected even after explaining all the benefits-P19

Some adolescents will agree because their peers had done it-P20

Some adolescents who are keen to learn something new will accept their blood and urine collected-P22

Some people will be afraid of the needle and that can prevent them from joining-P24

Some adolescents may consider it an opportunity to test their blood and that can encourage them to participate-P17

Many adolescents will be more willing to provide blood than urine samples-P19 and P22. If some one lacks blood that is a big consequence and this will make check for it-P22. Some may provide urine sample because they have ailments related to their urine like when they have painful urination-P17

- **Participants access to mobile phones**

Five of us have mobile phone and three of us don’t have one. Some of our parents does not allow us to use mobile when going to school especially the younger ones less than 15 years-P17-P24

- **Best network**

Africell-P17 Gamcell-P18 Q-cell- P19 Africell- P20-P24

- **Can network quality accommodate conversation**

It depends, sometimes the network is very poor and sometimes is good-P19

- **Alternative method to meet participants**

You can contact the focal person or meet participant in person-P21

Use social media like the radio to get access to the participants-P23

You can call some of the participants their parents to get access to them-P18

You can contact some participants siblings to get to the participants-P20

If communication on the phone is not possible, you should go back in person-P17

You can also use a focal person to gather the information on behalf of the researcher-P19

- **Completing the questionnaire**

The questionnaires were generally not badP17-P24

**Focus Group Discussion (FGD4)- Urban area II**

- **Cultural norms and practices**
- **What are the cultural norms and practices?**

Adolescents are told to reduce eating sour foods because it causes anaemia-P32

If a woman is pregnant, they are asked not to eat bitter tomatoes because it will make the child dumb-P29

A pregnant woman should not eat bitter tomatoes because it will cause skin rashes when the baby is born-P25

Adolescents should not eat pepper and sour foods because is not good for them-P28

Adolescents should not eat bitter things because it can move blood and water out of their body-P31

When adolescents are eating with elders, they should not put their hands at the middle of the bowl where the meat or fish is normally placed. They should wait for the elder to put the ingredients for them-P30

When a child is eating with elders, they should not look up but rather look down at the bowl until they are full and get up-P26

Adolescents should not join group of elders’ discussion-P29

When adolescents are eating with elders, they should wait until an elder touch the middle of the bowl first before they touch anything in the middle and their other hand should be holding the bowl whilst eating-P32

When a child is eating with elders, they should not talk-P31

School going adolescents should not eat the head of a catfish or head of fish in general because it will make them dull in school and they will not have good grades-P25 and P26

If someone does not eat bitter tomato, then that person is a witch or wizard-P25

- **How does these cultural norms and practices influence nutritional level of adolescents?**

Not allowing children to touch the ingredients at the middle of the bowl can cause malnutrition -P25-P32. Some elders are greedy, and they may even forget to put ingredients for the child-P32

Not allowing adolescents eat head of fish will not affect their nutritional status-P25-P32

Not eating bitter tomatoes does not influence one’s nutritional status-P30. Some people say eating bitter tomato is good because it increases fluids in their body-P32

Focusing where they are eating is good because it will help them eat well and they will know what ingredients to take-P32. Focusing in what you are eating when with others is good because it will prevent others not to put poison on your side whilst eating-P30

- **Which of the cultural norms and practices are beneficial?**

Focusing on the bowl when eating-P25-P32

- **Which of the cultural norms and practices are harmful?**

Not allowing children to touch the ingredients at the middle of the bowl-P25-P32

- **Views on diet, underweight and overweight**

Nutritional health refers to hypertension-P25 and P27

Nutritional health means healthy food-P26 and P28

Nutritional health refers to eating healthy food that makes one’s body healthy-P32

It means eating healthy food that prevent sickness-P31

It means someone should eat healthy food-P29

It refers to the food you eat that give you healthy living-PP30

- **Reasons for underweight**

It means the person is not eating food like foods that will give you energy like carbohydrates, proteins and vitamins-P30

That person does not eat healthy foods like always eating sour food that can cause ulcer-P31

If you are not eating healthy food, you will be underweight-P26

If they are not eating good food, they will be underweight-P27

They will be underweight if they are not eating healthy foods-P28

- **Prevention of underweight**

They should stop eating all the sour foods-P31

They should eat lot of proteins and vitamins-P30

Adolescents should eat good food like carbohydrate that will give them good weight-P32

Adolescents should eat energy giving foods like proteins and stop eating lot of sugar-P29

They should stop eating sour foods-P25

- **Reasons for overweight/obesity**

Overweight and obesity is caused by eating healthy foods-P26

It is caused by not eating healthy foods-P27

Eating rice and protein causes overweight and obesity-P28

Eating and sleeping too much and sitting at one place when you are in the house can lead to overweight and obesity-P25

By eating too much oil and sugary foods can cause overweight and obesity-P29

Sometimes fatness is a natural thing, but some people are also fat because they are sick-P32

You know some people at night when they eat dinner, they eat too much fat and immediately go to bed which can cause overweight and obesity-P29

They eat too much fats and oils which can cause overweight and obesity-P31

- **Prevention of overweight/obesity**

They should stop eating fats and oils because eating too much of fats and oils causes overweight and obesity-P31

They can also eat moderately not too much-P30

They should avoid eating too much and engage in exercise but sitting at one place is also a sickness on its own-P32

Stop eating certain foods that cause harm to their body like eating too much sugar-P29

Reduce eating too much food-P25 and P28

Stop eating too much oil and sugar and exercise everyday-P26

- **Composition of a healthy diet**

It comprises beans, cassava, potato and bread-P29

healthy diet comprises eating rice with little bit of salt added to it with vegetables such as cucumba, carrots and tomatoes-P31

It means eating rice and sauce like potato leaves by putting their little bit of salt, oil, maggi, pepper and palm oil-P30

It can consist of rice with little bit of salt together with meat soup-P32

Eating ‘Pemngbem’ is a nutritious diet because you don’t add oil in it and it has fish, lime, little bit of salt and rice-P25

Rice with meat stew together with vegetables like carrots, bitter tomatoes, egg plants and ‘supermeh’ is a nutritious diet-P28

Eating ‘Mbahal is healthy diet when it has fish, groundnut powder, rice, Jumbo and salt-P27

Eating fruits and vegetables is a healthy diet-P26

- **Perceived weight status**

Normal-P26 Normal-P27 Normal-P28 underweight-P28 Normal-P29

Normal-P32 Normal-P30 Underweight-P31

- **Views on addressing female adolescent malnutrition**
- **Undernutrition- National level**

The government should provide healthy foods for adolescents and tell parents not to be cooking unhealthy foods in the house-PP31

The government should give information regarding healthy foods to parents who in turn can advised their children about eating healthy foods-P30

Some people cook unhealthy food in their homes because that what they can afford, so the government should provide food for poor households and information for all households either rich or poor-P32

Government should provide adolescents with food and medical facilities-P29

The government should provide foods for adolescents by giving their parents money-P28

Government should sponsor poor households and the rich households also should help the poor households-P26

The government should provide television for some households so that parents are able to watch nutrition programs like cooking episodes and this can help them give good nutrition advise to their children-P30

- **Undernutrition-Community level**

Alkali’s can arrange meetings to discuss with members about nutrition issues-P31

Community leaders can call parents and talk to them about nutrition-P30

Community leaders can conduct a meeting with members and put in strategies to engage the government in supporting the community about arising nutritional issues-P32

Community leaders should organise a group and inform parents to feed children well-P29

Parents should be advised by community leaders the importance of cleanliness when cooking because some people pay attention to this when preparing food-P25

- **Undernutrition- Household level**

Parents should provide healthy foods and clean water for their children to grow healthy-P26

They should provide adolescents with good food and water-P27

Parents should cook good food for the adolescents to eat-P28

When parents are cooking, they should be very focused because anything can drop in and cause problem for them-P32

- **Overnutrition- National level**

The government should provide adolescent with a health facility where they can check their nutrition status- P30 and P31

The government should help adolescents with medications that can reduce their weight-P32

Government should provide adolescents with medicines and advised their parents to give them good food-P29

Government should consult parents and discuss with them about healthy lifestyle for adolescents by advising them to prevent their children from eating and sleeping too much and engage more in exercise-P25

Government should advise parents to provide good nutrition for their children-P28

Government should advise parents to provide good nutrition for their children-P28

- **Overnutrition-Community level**

Community leaders should communicate to parents about good nutrition who in turn can advise their children. The chiefs/Alkali’s should also collaborate with researcher and nutrition experts to advise adolescent on good nutrition-P31

Community leaders should discuss with parents and together put in measures to help adolescents-P27

Community leaders should encourage nutrition sessions among adolescents in schools and in the community through collaboration with nutrition and health experts-P32

Community leaders should communicate to parents to cook strong and healthy foods for their children which will help them grow-P26

They should provide good food and medications for adolescents-P27

They should provide good food for the adolescents-P28

- **Overnutrition- Household level**

Parents and guardians should take their children to the health centre for check up if they have nutrition issues-P30

They should take them to the hospital to be checked for nutritional problems-P31

Parents should be very observant about what their children, but some parents don’t pay attention to this at all. They should also make they get medical attention when they have nutrition issues-P32

Parents should be aware of their children eating habits-P29

- **Engaging adolescents in research**
- **Method:**

You should take permission from parents-P25

To give parents information sheets about the study so that they can allow their children to participate-P29

It is possible to recruit from madrassa or schools-P25

- **How to implement above method:**

You should meet parents in their homes and discuss with them the possibility for their children to participate in the study-P26, P27 and P28

Visit schools and madarasas-P25

- **Other methods**
- **Access to participants in community:**

You can organise a meeting with parents to meet them and discuss about the research and select the ones who want their children to participate-P30

You can go to the Alkalo or community chief to gain access to the community-P31

Recruiting from schools will be easier than house to house visit-P25-P32

- **Challenges to recruit participants:**

Some principals in schools may accept and others may reject-P30

Some parents may agree, and others may say no-P26 and P27

Some adolescents will not participate even if they are told by their parents to participate and if they are forced to participate, they may not be engaged in the discussion-P32

Some may accept and some may reject by either the parents or adolescents-P25-P32

- **Motivation factors-Parents:**

You know there are some parents they act before they think. They may reject even without reading the paper. In that case you should take your time and explain to them but when they insisted then you should leave-P31 and P32

- **Motivation factors-Participants**

Adolescents should discuss about the research with their parents so that they can come to terms regarding participating-P30

You should talk to the adolescent and try to convince them by telling them that it is important, and it can help them in the future-P30 and P31

Talk to them and tell them the relevance of it-P32

Discuss with adolescent parents-P29

You should provide gifts like reading books for adolescents because when they know that they will get something in return for participating, they will be very enthusiastic about it-P26

You should also provide additional gifts to those who participate well because that will make others to become very engage in the process-P32

- **Willingness to provide blood and urine sample**

Some adolescents may agree but some will not because they will think that you are going to inject them, but you can try and convince them-P31

Some will accept to participate but some will not-P30

Some adolescents may agree but some may not agree but you can talk to their parents about the importance and their parents can bring them to participate-P32

They will be willing to come because they want to learn about health issues-P29

If you tell adolescent you will give them gifts and take them to the supermarket, they will be willing to participate-P26

Some adolescents will not want to participate because they will think you will use their urine or blood for something else-P28

If you provide gifts to the adolescent, that will motivate them to participate-P25

- **Participants access to mobile phones**

Two participants have mobile, and 6 participants did not have a mobile phone

Many adolescents will not have their own mobile phones, but you can take the mobile number of their mothers if they agree to participate-P32

Most adolescents will not have access to mobile phones, but you can come in person again for follow up if communicating by phone is not possible-P30

Adolescents will not have mobile, but you can come back in person for follow up-P26 and P27

If they do not have personal mobile, you can call their mother to get them-P28

- **Best network**

Qcell-P31 Africell-P30 Qcell-P32 Qcell-P29 Africell-P25 Qcell-P28

Qcell-P27 Africell-P26

- **Can network quality accommodate conversation**

The network quality depends on the area the participant is**-** P25-P32

It is possible to get good quality-P30

It depends because sometimes the network is good but sometimes is poor-P32

The network will be good-P29

Voice message will be better-P25

I don’t know whether the network will be good because I don’t have access to mobile-P28

I don’t think the network quality will be good-P26

- **Alternative method to meet participants**

Many adolescents will not have their own mobile phones, but you can take the mobile number of their mothers if they agree to participate-P32

Most adolescents will not have access to mobile phones, but you can come in person again for follow up if communicating by phone is not possible-P30

Adolescents will not have mobile, but you can come back in person for follow up-P26 and P27

If they do not have personal mobile, you can call their mother to get them-P28

- **Completing the questionnaire**

Questionnaire was not difficult to answer-P25-P32
