# Supplementary material for: Factors influencing malnutrition among adolescent girls in The Gambia: a mixed-methods exploratory study
Source: BMC Public Health. 2025 Jan 8;25:80. doi: 10.1186/s12889-024-21242-w (PMC11708179; doi:10.1186/s12889-024-21242-w)
Supplement: Supplementary file 5 — Supplementary Material 5. Cut points for nutritional status categories [file 12889_2024_21242_MOESM5_ESM.docx]

**Cut points for nutritional status categories**

| **Nutritional status indices** | **Age group, years** | **Definition/ cut-points** | **Reference** |
| --- | --- | --- | --- |
| **z-scores** |  |  |  |
| BMI z scores | 5-19 | Individually assigned age-specific z-scores (mid-point of 6 months for each year) | World Health Organisation (WHO). World Health Organisation. Growth reference data for 5-19 years: World Health Organisation. [Webpage]. Available from: <https://www.who.int/tools/growth-reference-data-for-5to19-years> WHO; 2007 [Accessed: 11/7/24]. |
| Height for age z-scores | 5-19 | Individually assigned age-specific z-scores (mid-point of 6 months for each year) | WHO [As above] |
| **Weight status categories** |  |  |  |
| Underweight defined by BMI z scores | 5-19 | ≤−2  (according to age specific cut points) | WHO [As above] |
| Normal weight defined by BMI z scores |  | >−2 to <+1  (according to age specific cut points) | WHO [As above] |
| Overweight/ obese defined by BMI z scores | 5-19 | ≥+1SD to ≥+3SD  (according to age specific cut points) | WHO [As above] |
| Underweight defined by mid upper arm circumference (cm) | 10-14 | <21cm | Sethi V, Gupta N, Pedgaonkar S, Saraswat A, Dinachandra Singh K, Rahman HU, et al. Mid-upper arm circumference cut-offs for screening thinness and severe thinness in Indian adolescent girls aged 10-19 years in field settings. Public Health Nutrition. 2019;22(12):2189-99. |
| Underweight defined by Mid upper arm circumference (cm) | 15-19 | ≤22·6 cm | Sisay BG, Haile D, Hassen HY, Gebreyesus SH. Performance of mid-upper arm circumference as a screening tool for identifying adolescents with overweight and obesity. PLoS ONE. 2020;15(6):e0235063. |
| Normal weight defined by mid upper arm circumference (cm) | 10-14 | 21-22.9 | Defined by inference as the range between underweight and overweight |
| Normal weight defined by mid upper arm circumference (cm) | 15-19 | 22.7 27.8 | Defined by inference as the range between underweight and overweight |
| Overweight/ obese defined by mid upper arm circumference (cm) | 10-14 | >23 | Craig E, Bland R, Ndirangu J, Reilly JJ. Use of mid-upper arm circumference for determining overweight and overfatness in children and adolescents. Arch Dis Child. 2014;99(8):763-6. |
| Overweight/ obese defined by mid upper arm circumference (cm) | 15-19 | ≥27.9 | Sisay *et al* [As above] |
| Abdominal obesity defined by waist circumference (cm) | 7-17 | ≥71.2 | Daniels SR, Khoury PR, Morrison JA. Utility of different measures of body fat distribution in children and adolescents. American Journal of Epidemiology. 2000;152:1179-84. |
| Abdominal obesity defined by waist: hip ratio (cm) | 7-17 | ≥0.82 | Daniels *et al* [As above] |
| **Stunting** |  |  |  |
| Stunting defined by height for age z-scores | 5-19 | ≤−2 SD (according to age specific cut points) | WHO [As above] |
