# Supplementary material for: Factors influencing malnutrition among adolescent girls in The Gambia: a mixed-methods exploratory study
Source: BMC Public Health. 2025 Jan 8;25:80. doi: 10.1186/s12889-024-21242-w (PMC11708179; doi:10.1186/s12889-024-21242-w)
Supplement: Supplementary file 6 — Supplementary Material 6. Questionnaire results [file 12889_2024_21242_MOESM6_ESM.docx]

**Individual, household and community-level factors, by setting**

|  | **N (%)^1^** | | | **P-value*** |
| --- | --- | --- | --- | --- |
| **Individual-level variable** | **All** | **Urban** | **Rural** |  |
| **Had malaria** |  |  |  |  |
| Never | 17 (53.1) | 5 (31.3) | 12 (75.0) |  |
| Ever | 15 (46.9) | 11 (68.8) | 4 (25.1) |  |
|  |  |  |  | **0.013** |
| **Received treatment for parasitic worms** | |  |  |  |
| Never | 21 (65.6) | 9 (56.3) | 12 (75.0) |  |
| Ever | 11 (34.4) | 7 (43.8) | 4 (25.0) |  |
|  |  |  |  | 0.264 |
| **Access to nutrition information** |  |  |  |  |
| Adequate | 8 (25.0) | 8 (50.0) | 0 (0.0) | **<0.0001** |
| **Physical activity** |  |  |  |  |
| Adequate | 12 (37.5) | 6 (37.5) | 6 (37.5) | 1.000 |
| **Access to a mobile phone** |  |  |  |  |
| Yes | 12 (37.5) | 8 (50) | 4 (25) | 0.14 |
| **Watch television** |  |  |  |  |
| Yes | 17 (53.1) | 13 (81.2) | 4 (25.0) | **0.008** |
| **Use of the internet** |  |  |  |  |
| Yes | 10 (31.2) | 10 (62.5) | 0(0.0) | **0.002** |
| **Sleep duration** |  |  |  |  |
| Adequate | 20 (62.5) | 15 (93.8) | 5 (31.3) | **<0.001** |
| **Household factors** |  |  |  |  |
| **Family type** |  |  |  |  |
| Extended | 21 (65.6) | 7 (43.8) | 14 (87.5) | **0.009** |
| **Number of siblings** |  |  |  |  |
| Up to 4 | 13 (40.6) | 9 (56.3) | 4 (25.0) |  |
| 5 or more | 19 (59.4) | 7 (43.8) | 12 (75.0) |  |
|  |  |  |  | 0.053 |
| **Home ownership** |  |  |  |  |
| Home owned, Yes | 25 (78.1) | 13 (81.3) | 12 (75.0) | 0.669 |
| **Homeowner [among those whose homes were owned]** | | |  |  |
| Father | 11 (44.0) | 5 (38.5) | 6 (50.0) |  |
| Family member/friend | 14 (56.0) | 8 (61.5) | 6 (50.0) |  |
|  |  |  |  | 0.561 |
| **Mother’s occupation** |  |  |  |  |
| Paid work | 20 (62.5) | 8 (50.0) | 12 (75.0) |  |
| Unpaid work/ carer/ not working/ deceased | 12 (37.5) | 8 (50.0) | 4 (25.0) |  |
|  |  |  |  | 0.08 |
| **Father’s occupation** |  |  |  |  |
| Paid work | 17 (54.8) | 9 (60.0) | 8 (50.0) |  |
| Unpaid work/ carer/ not working/ deceased | 14 (45.1) | 6 (40.0) | 8 (50.0) |  |
|  |  |  |  | 0.232 |
| **Mother’s education** |  |  |  |  |
| No formal education | 17 (53.1) | 8 (50.0) | 9 (56.3) |  |
| At least primary school | 15 (46.9) | 8 (50.0) | 7 (43.8) |  |
|  |  |  |  | 0.723 |
| **Father’s education** |  |  |  |  |
| No formal education | 16 (50.0) | 5 (31.3) | 11 (68.7) |  |
| At least primary school | 16 (50.0) | 11 (68.7) | 5 (31.3) |  |
|  |  |  |  | **0.034** |
| **Drinking water source** |  |  |  |  |
| Tap | 28 (87.5) | 16 (100.0) | 12 (75.0) | 0.102 |
| **Shop for household food in the community** |  |  |  |  |
| Yes | 29 (90.6) | 13 (81.3) | 16 (100.0) | 0.069 |
| **Electricity in household** |  |  |  |  |
| Yes | 18 (56.3) | 13 (81.3) | 5 (31.3) | **0.004** |
| **Electricity type in household [among those with electricity]** | | |  |  |
| National supply | 12 (66.7) | 12 (75) | 0 (0.0) | **0.010** |
| **Community factors** |  |  |  |  |
| **Access to a range of food markets** | |  |  |  |
| 1 or 2 markets | 16 (50.0) | 0 (0.0) | 16 (100.0) |  |
| >3 markets | 16 (50.0) | 16 (100.0) | 0 (0.0) |  |
|  |  |  |  | **<0.001** |
| **Healthcare services** |  |  |  |  |
| None | 6 (18.8) | 0 (0.0) | 6 (37.5) |  |
| Conventional (hospital, clinic, pharmacy) | 16 (50.0) | 16 (100.0) | 0 (0.0) |  |
| Traditional (midwife) | 10 (31.3) | 0 (0.0) | 10 (62.5) |  |
|  |  |  |  | **<0.001** |
| **Road quality** |  |  |  |  |
| Standard (Asphalt and concrete) | 16 (50.0) | 16 (100.0) | 0 (0.0) |  |
| Gravel | 6 (18.8) | 0 (0.0) | 6 (37.5) |  |
| No pavements | 10 (31.3) | 0 (0.0) | 10 (62.5) |  |
|  |  |  |  | **<0.001** |
| **Electricity type in community** | |  |  |  |
| National supply, Yes | 16 (50.0) | 16 (100.0) | 0 (0.0) | **<0.001** |
| **Travel to attend school** |  |  |  |  |
| Yes | 12 (37.5) | 5 (31.3) | 7 (43.8) | 0.465 |
| **Reason for travelling to school [among those who travel to attend school]** | | | |  |
| No school in area | 7 | 0 (0.00) | 7 (100.0) |  |
| Other | 5 | 5 (100.0) | 0 (0.00) |  |
|  |  |  |  | **0.001** |

^1^N (%) only reported for those categories within variables where n=>4. *For differences between urban and rural samples, significant p-values (p<0.05) are in bold type
